# Medial knee loading is altered in subjects with early osteoarthritis during gait but not during step-up-and-over task

**DOI:** 10.1371/journal.pone.0187583

**Published:** 2017-11-08

**Authors:** Susana Meireles, Mariska Wesseling, Colin R. Smith, Darryl G. Thelen, Sabine Verschueren, Ilse Jonkers

**Affiliations:** 1 Department of Kinesiology, Katholieke Universiteit Leuven, Leuven, Belgium; 2 Department of Mechanical Engineering, University of Wisconsin-Madison, Madison, Wisconsin, United States of America; 3 Department of Rehabilitation Sciences, Katholieke Universiteit Leuven, Leuven, Belgium; University of Memphis, UNITED STATES

## Abstract

This study evaluates knee joint loading during gait and step-up-and-over tasks in control subjects, subjects with early knee OA and those with established knee OA. Thirty-seven subjects with varying degrees of medial compartment knee OA severity (eighteen with early OA and sixteen with established OA), and nineteen healthy controls performed gait and step-up-and-over tasks. Knee joint moments, contact forces (KCF), the magnitude of contact pressures and center of pressure (CoP) location were analyzed for the three groups for both activities using a multi-body knee model with articular cartilage contact, 14 ligaments, and six degrees of freedom tibiofemoral and patellofemoral joints. During gait, the first peak of the medial KCF was significantly higher for patients with early knee OA (*p* = 0.048) and established knee OA (*p* = 0.001) compared to control subjects. Furthermore, the medial contact pressure magnitudes and CoP location were significantly different in both groups of patients compared to controls. Knee rotation moments (KRMs) and external rotation angles were significantly higher during early stance in both patient groups (*p* < 0.0001) compared to controls. During step-up-and-over, there was a high variability between the participants and no significant differences in KCF were observed between the groups. Knee joint loading and kinematics were found to be altered in patients with early knee OA only during gait. This is an indication that an excessive medial KCF and altered loading location, observed in these patients, is a contributor to early progression of knee OA.

## Introduction

Osteoarthritis (OA) is a chronic degenerative and multifactorial [1,2] joint disease that most frequently affects the knee [3], causing pain and functional disability. To date, there are no therapeutic interventions that overcome or effectively delay the progression of this disease and symptoms can only be managed [4]. Identifying the contributing factors associated with early stages of OA is imperative to classify patients at high risk to develop established knee OA and better assess effective treatments to protect joint integrity before major structural damage occurs.

Although the cause of OA is still not completely understood, biomechanical factors are known to play an important role [5,6]. Aberrant knee joint loading has been identified as a factor affecting the progression of knee OA [7–9]. External joint moments can be readily calculated from motion analysis data and thus have been proposed to identify characteristics of OA patients. Elevated knee adduction moment (KAM), the external knee joint moment in the frontal plane, has been used as a parameter reflecting elevated medial tibiofemoral loading [10–17] and was associated with the presence of medial knee OA [18]. Reduced external knee flexion moment (KFM), the external knee joint moment in the sagittal plane, is commonly reported for OA patients as a consequence of quadriceps weakness [19–22]. However, some studies in patients with early stages of knee OA suggest that altered KAM and KFM are not risk factors in the initial development of knee OA [16,17,23,24]. Only a few studies examined the external knee rotation moment (KRM), the external moment in transverse plane, for patients with knee OA and they report contradictory findings of altered KRM [19,25–28] in patients with OA compared to healthy subjects. In addition, for KRM, no comparison between early and established OA patients is available to date. Consequently, the ability of external joint moments to identify the onset of OA is still under debate [17].

Knee joint moments depend only on kinematics and external forces and, therefore, do not account for muscle forces. Consequently, a reduction in peaks KAM does not necessarily indicate a reduction in medial contact load [14]. On the other hand, knee contact force (KCF), calculated using musculoskeletal modeling in combination with dynamic simulations, directly reflect cartilage loading by accounting for muscle and ligament forces.

A previous study from our group showed that in early stages of knee OA, overall KCFs were not different from those in control subjects [23], but were more elevated in subjects with established OA. By differentiating the loading on the medial and lateral compartment, Kumar *et al*. [16] found higher medial KCFs in patients with established OA (with Kellgren-Lawrence score (K&L) ≥ 2) than in healthy subjects. Marouane et al. [29], have recently reported KCFs and their respective location during the stance phase in both healthy subjects and subjects with established knee OA (K&L = 3 or 4) aiming to compare various approaches to compute the KCF locations in both groups. This study focused on subjects with demonstrated radiographic knee OA. Therefore, to date no information on the medio-lateral load distribution in terms of KCFs and/or alterations in contact location of loading in the joint are available in early OA patients. Alterations in cartilage surface contact location have been suggested to occur during gait and associated to the high incidence of medial knee OA after anterior cruciate ligament (ACL) injury [30]. Interestingly, advances in musculoskeletal modeling now enable evaluation of the pressure distribution in the joint and therefore can provide insight into the load-bearing regions of the knee joint [31,32]. As such, shifts in contact location during weight-bearing activities can be evaluated, an action mechanism often suggested to contribute to the onset of OA [2].

Most studies in literature have focused on knee loading during gait as a biomarker for OA onset and progression. However, subjects with knee OA initially present pain complaints in more demanding tasks, specifically weight-bearing activities that involve large knee flexion [33]. There are only a few studies that have reported joint moments [33–37] and muscle activations [38] during stair negotiation in patients with severe knee OA. Similar to stair negotiation (walking on a flight of stairs/steps), step-up-and-over requires an upward propulsive phase (as stair ascent) as well as a downward energy absorption phase (as stair descent), recruiting large knee motion and high muscle force. Studies have shown lower KFM [33,37] and indications of lower KAM during stair ascent and descent [35] in patients with knee OA compared to healthy subjects. So far, compartmental KCFs have not been described in patients with knee OA during higher demanding tasks. However, these metrics are extremely relevant, as demanding movements might explain mechanical alterations earlier and therefore may be more helpful in identifying early OA, enabling earlier screening and treatment.

The first aim of this study is to evaluate the magnitude of knee joint loading (KCF in the medial and lateral knee compartment, maximum contact pressures and centre of pressure locations in the medial compartment) during gait in patients with early knee OA, and those with established knee OA compared to healthy subjects. We hypothesize that these parameters are more helpful in detecting early altered knee joint loading, prior to the onset of structural degeneration. Secondly, this study evaluates knee joint loading during step-up-and-over task in early OA subjects. We hypothesize that this higher demanding activity may already cause larger alterations in the medial compartment loading, present prior to alterations during gait and, therefore, may be able to discriminate patients with early knee OA from healthy subjects.

## Methods

The current study represents an extended analysis of the data set reported in [23].

### Participants

Fifty-three participants (all women, mean age of 64.8±7.5, from 37 to 78 years) were recruited and separated into three groups: asymptomatic healthy subjects (*n* = 19) as control; patients with symptomatic early medial knee OA (*n* = 18), and patients with symptomatic established medial knee OA (*n* = 16). Participants were recruited between 2008 and 2011. Patients with OA were recruited by a rheumatologist or orthopedic surgeon during weekly consultations in the University Hospitals Leuven. Healthy participants were recruited from cultural and social organizations. All procedures were approved by the local ethics committee of Biomedical Science, KU Leuven, Belgium. Written informed consent was obtained from each subject. The current analysis is part of a larger study already published by Baert *et al*. [39–41] and Mahmoudian *et al*.[42–44].

Early medial knee OA was diagnosed based on novel classification criteria of Luyten et al. [45], including fulfillment of three criteria, namely knee pain, a K&L [46] grade 0, 1 or 2− (osteophytes only) and structural changes observed on MRI.

Established medial knee OA was diagnosed based on slightly adapted American College of Rheumatology classification criteria [47], including knee pain, morning stiffness of less than 30 min duration and crepitus, together with structural changes defined as presence of minimum grade 2+ (osteophytes and joint space narrowing) on K&L scale for at least the medial compartment on radiography.

A control group was also analyzed, which included asymptomatic healthy subjects with no history of knee OA or other pathology involving any lower extremity joints, and with a radiological score of 0 or 1 according to K&L score.

Participants were excluded if they had a prior significant trauma or surgery in lower limbs and/or low back, if they suffered from a neurological disease affecting coordination and/or balance during gait and/or musculoskeletal disorders other than knee OA in one of the limbs during the last six months prior to testing.

Subject characteristics are listed in Table 1. Knee pain was assessed through the Knee Injury and Osteoarthritis Outcome Score (KOOS) (Dutch version [48]). Knee joint alignment in the frontal plane was measured by a single experienced observer on full-leg, anterior-posterior, weight-bearing radiographs of the lower limbs (Oldelft, Triathlon, Agfa ADC M Compact Plus) [49].

**Table 1:** Characteristics of the groups. Control (CO), early OA (EA) and established OA (ES).

	Task	Control	Early OA	Established OA	*p*	*p* (CO-EA)	*p* (CO- ES)	*p* (EA- ES)
No. of subjects	Gait	17	14 (6uni+8bi)	16 (16bi)	*-*	*-*	*-*	*-*
	Step	19	18 (8uni+19bi)	16 (16bi)	*-*	*-*	*-*	*-*
Age, *years* (age range)	Gait	64.2±9.0 (37 to 78)	63.3±7.7 (49 to 73)	67.2±6.7 (54 to 78)	0.362	0.985	0.619	0.449
	Step	64.3±8.5 (37 to 78)	63.3±7.0 (49 to 73)	67.2±6.7 (54 to 78)	0.305	0.965	0.598	0.351
Body mass, *kg*	Gait	64.0±7.9	69.7±16.6	73.3±11.9	0.103	0.494	0.102	0.809
	Step	64.6±7.7	70.0±15.5	73.3±12.0	0.103	0.440	0.109	0.813
Height, *m*	Gait	1.61±0.1	1.62±0.1	1.61±0.1	0.828	0.971	0.993	0.903
	Step	1.62±0.1	1.62±0.1	1.61±0.1	0.837	0.994	0.974	0.910
KOOS pain score	Gait	100±0.0	82.9±17.7	73.3±19.4	**0.000**	**0.005**	**0.000**	0.203
	Step	100±0.0	84.4±15.4	73.4±19.4	**0.000**	**0.004**	**0.000**	0.075
Speed, *m/s*	Gait	1.21±0.2	1.26±0.2	1.20±0.2	0.426	0.623	0.992	0.524
	Step	0.53±0.1	0.55±0.1	0.57±0.1	0.311	0.663	0.371	0.966
Knee Alignment in the frontal plane °	Gait	0.50±2.3 (24)	1.46±3.4 (15)	3.66±3.5 (13)	**0.014**	0.701	**0.010**	0.164
	Step	0.45±2.5 (26)	1.14±3.2 (18)	4.03±3.5 (12)	**0.004**	0.831	**0.003**	**0.034**

Values are the mean ± Standard Deviation (SD). ANOVA with Gabriel *post hoc* test. Significant difference *p <* 0.05 are indicated in bold.

For the knee alignment, positive values indicate varus (adduction) alignment and negative values indicate valgus (abduction) alignment.

*Uni* corresponds to the number of patients with unilateral OA and *bi* to those with bilateral OA.

**Fig 1:**
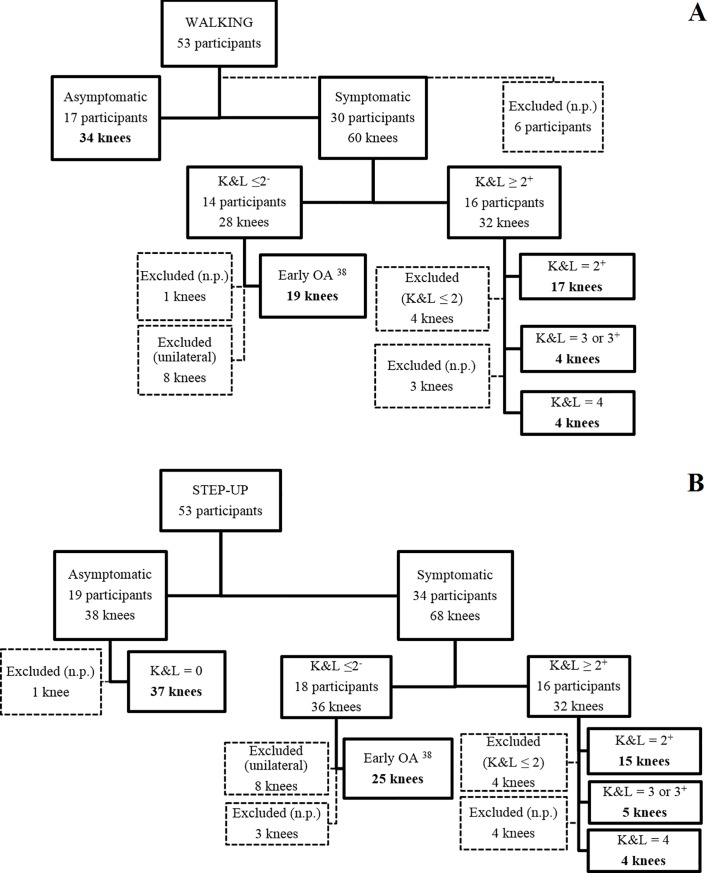
**Flow charts of the limbs selection for gait (A) and step-up-and–over (B).** The final number of the analyzed limbs are indicated in bold. During gait, 11%, 50% and 7% of the total knees diagnosed with early OA presented K&L of 0, 1 and 2, respectively. During step-up and -over, 17%, 47% and 6% of the total knees diagnosed with early OA presented K&L of 0, 1 and 2, respectively. Numerical problems are indicated as *n*.*p*.

For healthy subjects, both legs were analyzed. For symptomatic patients with unilateral knee OA, only data of the affected knee were analyzed. For those with bilateral knee OA, both legs were analyzed except if the less involved side presented with a K&L score ≤ 2 (Fig 1 and Table 1) for the established OA group.

All recruited subjects performed gait and step-up-and-over tasks. However, due to numerical problems during the simulation, six subjects were excluded from the gait analysis (Fig 1). More details about the participants’ selection and the total number of limbs included in each group are presented in Fig 1.

### Motion analysis

An active 3D motion analysis system (Krypton, Metris) recorded the 3D position of 27 LEDs at a sampling frequency of 100 Hz placed according to an extended Helen Hayes protocol (consisting of 5 technical clusters and 12 anatomical landmarks). Marker data were labeled and smoothed using a spline routine [50] in Matlab R2010b (Mathworks, inc.) with cut-off at 6Hz. A force plate (Bertec Corporation, USA) measured ground reaction forces (GRF) sampled at 1000 Hz. GRF were filtered using a second order Butterworth low pass filter, with cut-off level at 30Hz.

Gait analysis consisted of barefoot level walking along a 10 m walkway at self-selected speed with the force plate embedded in the middle of the walkway. Subjects were required to perform 6 trials for each leg.

**Fig 2:**
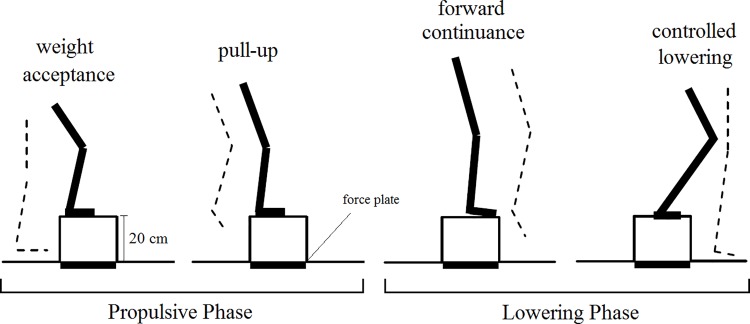
A schematic illustrating the step-up-and-over task (adapted from Reid [52]). The stepping leg (bold) is the leg considered for further analysis.

Step-up-and–over analysis consisted of barefoot [51] stepping onto a 20-cm-high step with one leg (stepping leg), while stepping over with the other leg (trailing leg) making contact on the other side of the step (Fig 2). The force plate was embedded in the ground under the step. Subjects performed a total of 3 trials for each leg.

### Musculoskeletal model

**Fig 3:**
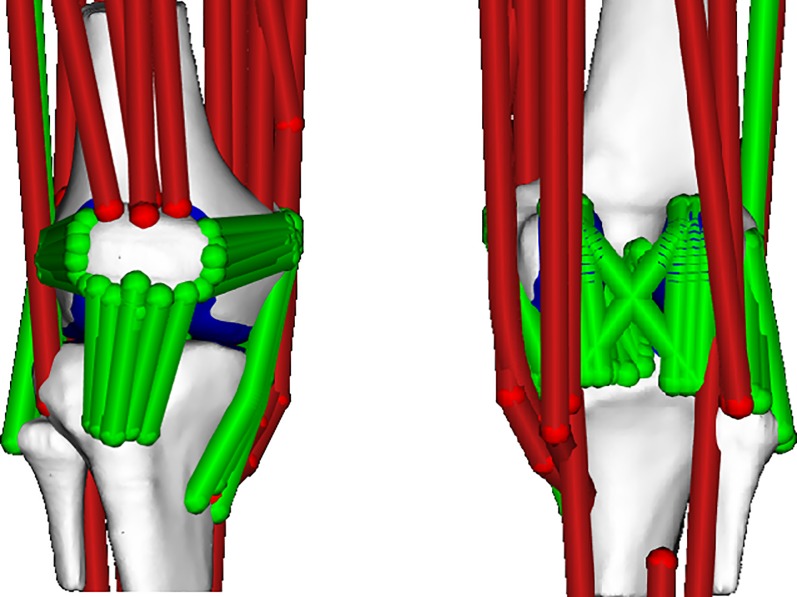
Multibody 12 degree of freedom knee model [53] including ligaments and an elastic foundation contact model.

A multi-body knee model (Fig 3) with 6 degrees of freedom for the tibiofemoral and patellofemoral joints was used [53]. Fourteen ligaments were represented by bundles of nonlinear elastic springs. Cartilage surface contact pressures were computed using an elastic foundation formulation [54]. The knee model was integrated into an existing lower extremity musculoskeletal model [55], which included 44 musculotendon units crossing the hip, knee and ankle joints.

The lower extremity model was scaled to subject-specific segment lengths as determined in a static calibration trial. The joint angles were computed using an inverse kinematics algorithm. The concurrent optimization of muscle activations and kinematics (COMAK) algorithm [31], was used to compute the secondary tibiofemoral (tibiofemoral translations and non-sagittal rotations) and patellofemoral kinematics, muscle and ligament forces, and KCFs by minimizing the muscle volume weighted sum of squared muscle activations plus the net knee contact energy. Tibiofemoral cartilage contact pressures were computed using a non-linear elastic foundation model in which pressure is assumed to be a function of the depth of penetration between meshes of the contacting cartilage surfaces. Depths of penetration for each triangle in a mesh were determined at each time step using ray-casting techniques [53]. At each triangle of the tibia plateau, the contact pressure was computed, in which cartilage was assumed to have an elastic modulus of 10 MPa, a Poisson's ratio of 0.45, and a uniform thickness of 2 mm for each surface (i.e. 4 mm total thickness) [54]. Subsequently, an inverse dynamics algorithm computed the external joint moments: KFM, KAM and KRM.

Total KCFs correspond to the resultant tibiofemoral contact forces in the entire joint, while medial and lateral KCFs correspond to contact forces in the medial and lateral compartment, respectively. KCFs are expressed in the tibia reference frame. Calculated KCFs were normalized to body weight (BW) and moments were normalized to the product of body weight and height (BW×Ht). All data were time normalized to the stance phase (i.e. from initial contact to toe off of the ipsilateral leg).

### Data analysis

During gait, KCFs, moments and angles throughout the stance phase were averaged over the 6 trials for each leg. The peaks during the first and second half of the stance phase were determined for the total KCF, medial KCF, and lateral KCF, KFM and KRM. The minimum total, medial and lateral KCF and KFM during the single support (SS) phase, corresponding to the middle of the stance phase, occurring from toe-off until heel strike of the contralateral foot, between 25% and 75% of the gait cycle, were determined. For the KAM, only the first peak during early stance, corresponding to the highest peak during stance, was calculated. Although two peaks in KAM have been reported for healthy subjects and patients at early stages of OA, patients with advanced medial knee OA frequently present one peak during early stance and, therefore, a minimum value during SS and a second peak were not always clear [17,56–59]. A similar trend was found in our study, in which some patients with established knee OA did not show a distinct second peak.

During step-up-and-over, KCFs, joint moments and angles of the stepping leg were averaged across the 3 trials throughout the stance phase for each leg. The maximal values of total KCF, lateral KCF, KFM and KAM during the first and second half of the stance phase and the minimum values during the SS phase were determined (minimum total KCF, minimum lateral KCF, minimum KFM, and minimum KAM, respectively). In addition, the highest peak medial KCF during the stance phase was compared between groups. Due to the high variation in the individual KRM pattern observed in patients with established OA during this task, maximum values of KRM were not calculated and only the average curve is presented.

Furthermore, at the time instant of peak medial KCF, the maximum contact pressure magnitude and center of pressure (CoP) location in the medial tibial plateau were assessed and compared between the groups. The medial-lateral and anterior-posterior locations of CoP, calculated with respect to the tibia reference frame, were normalized to the tibia size and compared between the groups.

### Statistical analysis

One-way analysis of variance (ANOVA), performed with SPSS Inc., v17.0, evaluated whether differences in peaks and minimum moments, KCF, contact pressures and CoP location were significantly different (*p* ≤ 0.05) between the three groups. As sample sizes were slightly different, Gabriel *post hoc* test was used to assess whether the differences were significant.

The effect size (Cohen's *d*) on these ANOVA tests were evaluated using G*Power 3.1.9.2 [60] based on the assumption of less than 5% Type I error. The effect size (*f*, from population means) for the *F*-test ANOVA were considered small for *f* = 0.10, medium for *f* = 0.25 and large for *f* = 0.40 with a minimum effect size (*d*, from standard deviations) of 0.80 considered acceptable [61].

## Results

### Subject characteristics

Age, body mass, height, and speed for gait and step-up-and–over did not differ significantly between the three groups (Table 1). Both OA groups reported significantly greater knee pain (*p* < 0.001) than controls, but no difference was found between the two groups of OA patients. Patients with established OA presented significantly higher varus alignment compared to controls in both gait and step-up-and-over (*p* = 0.010 and *p* = 0.003, respectively).

### Knee joint loading during gait

**Fig 4:**
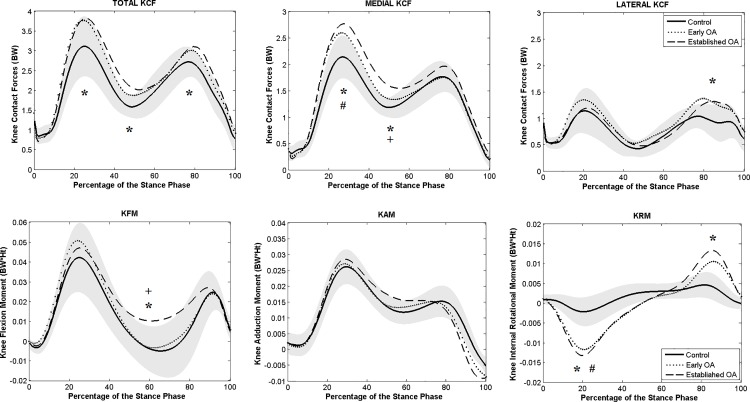
Averaged total, medial and lateral KCF (above) and knee moments (below) during stance phase of gait. Knee moments in the sagittal, frontal and transversal planes are presented. The gray shaded area corresponds to the standard deviation of the control group. ^*^ indicates a significant difference between established OA and control group. ^**#**^ indicates a significant difference between early OA and control group. ^**+**^ indicates a significant difference between the early and established OA.

Only patients with established knee OA showed significantly higher peaks and higher minimum total KCF during the single support phase (Fig 4), when compared to controls (*p* = 0.012 and *p* = 0.013 during both first and second peak and *p* < 0.0001 during SS). No significant difference in total KCF was found between early OA and control subjects.

Both patient groups presented higher peak medial KCF compared to controls (*p* = 0.001, established OA and *p* = 0.048, early OA). Lateral compartment KCFs were higher in both OA groups during the second part of stance compared to healthy subjects (S1 Table) but only significantly for the established knee OA group (*p* = 0.009).

**Fig 5:**
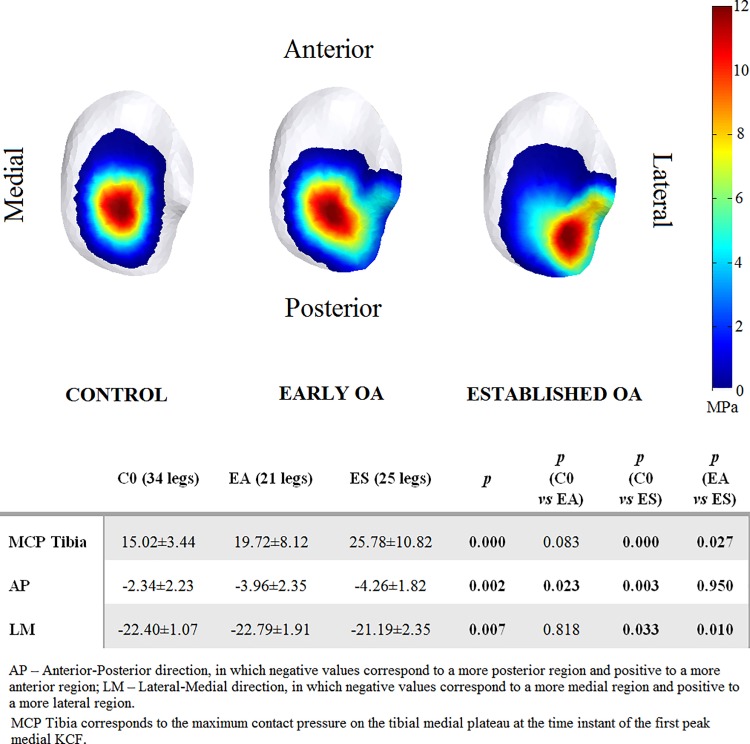
Group-averaged contact pressure distributions on the articular surfaces of medial tibial plateau at the time instant of the first peak medial KCF. To obtain these averaged contact pressure distribution maps, the average contact pressure was calculated for every triangle of the medial tibial surface mesh and presented on a representative surface model. Results are presented for the control group (C0, on the left), the early knee OA group (EA, in the middle), and the established knee OA group (ES, on the right).

In Fig 5, the average contact pressure distribution on the tibial plateau at the time instant of the first peak medial KCF are presented for the three groups. Maximum contact pressure was significantly higher for subjects with established OA (25.78±10.82 MPa) compared to the control (15.02±3.44 MPa) and early OA (19.72±8.12 MPa) groups. In subjects with early knee OA, the medial compartment CoP at the time instant of the first peak medial KCF was shifted from central (as seen in the control subjects) to a significantly more posterior region, while a significantly more postero-lateral location of the CoP was found in subjects with established OA.

No significant differences were found in peak KFM or KAM between the three groups. During SS, patients with established knee OA presented significantly higher KFM compared to control and early OA groups (S1 Table). First peak KRM was significantly higher in early (*p* < 0.0001) and established OA (*p* < 0.0001) groups compared to healthy subjects. Only established OA showed significantly higher second peak KRM (Fig 4).

At the time instant of the first peak medial KCF, the tibia was significantly more externally rotated with higher variation for both OA groups (rotation angle means and respective standard deviations of –7.4°±14.0° and –14.6°±14.3°, respectively, for early and established OA) compared to the controls (rotation angle mean and respective standard deviation of +0.3°±5.1°) (S1 Fig and S1 Table).

For all reported significant differences, the effect size *f* was large or medium to large (*f* ≥ 0.34) and *d* ranged from acceptable (*d* ≥ 0.80) to very high (*d* ≥ 0.95) (S1 Table). Only for the second peak KRM a *d* lower than 0.80 was found.

### Knee joint loading during step-up-and-over

**Fig 6:**
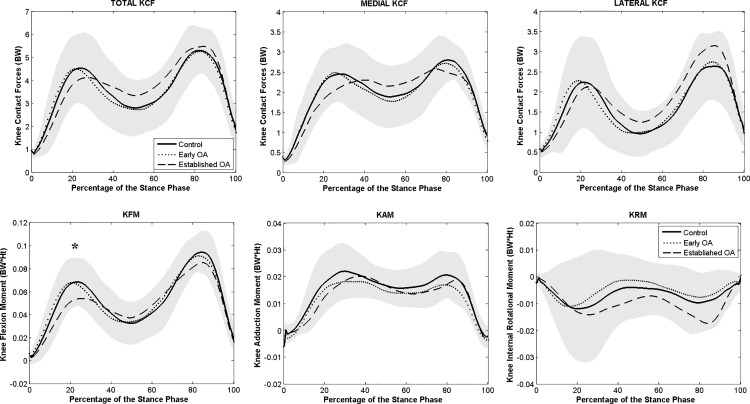
Averaged total, medial and lateral KCF (above) and knee moments (below) during stance phase of step-up-and-over. Knee moments in the sagittal, frontal and transversal planes are presented. The gray shaded area corresponds to the standard deviation of the control group. *indicates a significant difference between established OA and control groups.

No significant differences in KCF, either medial or lateral, were observed between the groups (Fig 6). Due to the high variability in terms of joint angles (S2 Fig), moments and KCFs (S2 Table) between subjects during the step-up-and-over task, *f* values were small to medium and *d* did not achieve the acceptable minimum. Therefore, the contact pressure data was not further analyzed.

Patients with established knee OA did present significantly lower first peak KFM compared to controls (*p* = 0.038) (S2 Table). No significant differences were observed in terms of KAM between the three groups (Fig 6).

## Discussion

This study investigated magnitude and location of knee loading during gait and step-up-and-over in subjects with early and established medial knee OA. We evaluated loading differences, both KCFs and contact pressure distributions, between patients with early OA compared to healthy subjects and patients with established OA aiming to identify whether early changes in knee loading are already present in these patients.

### Knee joint loading during gait

Our study shows that in patients at early stages of OA, altered medial compartment knee loading is found when using a musculoskeletal model which calculates compartmental joint loading. An elevated overall mechanical loading during gait, assessed by the total KCF, is only present in more advanced stages of the disease as observed in patients with established knee OA and not in early stages. Likewise, although the maximum contact pressures were more elevated in both groups of OA patients compared to controls, it was only significant in patients with established OA. This partially confirms our first hypothesis, in which medial KCF during gait showed to be helpful in detecting early changes in knee loading. Nevertheless, both patients groups with knee OA showed a shifted CoP at the first peak medial KCF which, in combination with higher external rotation of the tibia than healthy subjects during early stance, shows that patients with knee OA tend to load a more posterior (both groups) and lateral (established OA) cartilage region of the medial tibia plateau, which is not loaded in healthy subjects (Fig 5). This suggests that, although excessive loading is not revealed by the total KCF in the early phase of the disease, the medial-lateral force and pressure distributions are altered. Only when clear structural degeneration is present (KL>2), as in the established OA group, changes in gait mechanics result in excessive total knee loading compared to healthy controls. The abnormal transverse plane kinematics, particularly the more elevated external rotation found in patients with OA than in controls, shifted the normal load bearing contact to regions in the cartilage which are less predisposed for higher loads and therefore might influence the initiation of knee OA. If the normal load bearing contact has shifted to a region less predisposed for higher and repetitive loading during walking, due to ligament-deficiency or -injury, there is the potential for a degenerative pathway to be initiated [62].

In regards to the external joint moments during gait, only peak KRMs were significantly different between patients with early knee OA and healthy subjects. Patients with established OA showed higher midstance KFM, but no differences in peak KAM or KFM compared to the other two groups, confirming our previous study [23]. First peak KRM was higher in patients with knee OA than in healthy subjects. For patients with established OA, the excessive rotation moments persisted during late stance. Although KRM has received much less attention in the literature than KAM or KFM, and less consistence has been found between studies assessing patients with knee OA [19,25,26,63,64], our findings confirm the study of Gok *et al*. [25] and Wilson *et al*. [26], who found higher rotation moments in patients with knee OA compared to healthy subjects.

Comparing to previous literature, similar patterns of joint moments were observed in the present study [16,19,23,25,26,63,64]. In terms of magnitudes, studies show more differences in knee joint moments between each other, depending on the group of participants and techniques used to calculate knee moments. Magnitudes of peak KFM as well as moments in the other two planes of movement in healthy subjects and subjects with OA were in general comparable to those presented by Gok *et al*. [25] and Kumar *et al*. [16]. Significant differences in peak KAM have been previously reported [65–69] and are in contrast with the present study. Previous studies have used more simplified knee models, presenting less degrees of freedom and calculated knee kinematics using the transepicondylar axis (TEA), therefore not accounting for load-dependency effects on knee angle calculations. More simplified knee models can induce differences in the calculated kinematics and, ultimately, in KAM compared to more complex knee models. Firstly, large errors in the calculation of the secondary kinematics (varus-valgus angle) are expected when using TEA to calculate knee angles [70–74]. Secondly, by tracking the secondary kinematics, models do not take load-dependency effects into account [54,74–77] that may be relevant especially in patients with knee OA [74] as they normally present more joint instability [78]. Indeed, in a previous study [74], the effect of the knee axis on the calculated KAM was assessed, which underlined the sensitivity of KAM to knee axis definition. The current knee model includes six degrees of freedom in the tibiofemoral and six in the patellofemoral joints and accounts for load-dependent effects in the moment calculation that may explain the differences in angles and moments compared to previous studies.

The present study provides important insight into the altered medial loading magnitude and medial pressure location which were found to be already present in patients at early stages of medial knee OA, but was not revealed by the total KCF. With progression of structural degeneration, alterations in gait mechanics led to elevated overall joint loading, affecting both the medial and lateral compartment. Therefore, medial KCF rather than KAM or total KCF during gait provides the most helpful marker for early OA.

### Knee joint loading during step-up-and-over

High variations in movement strategies between subjects, particularly in those having knee OA, were observed during step-up-and-over. Due to these high variations, the effect size was low. Consequently, step-up-and-over does not generate large differences in kinematics and loading patterns, which might be due to the difficulty of standardizing the movement execution. As a more demanding task, step-up-and-over seems to motivate subjects, particularly those with knee OA, to search more for alternative movement strategies to deal with and, therefore, generating elevated variations.

In contrast to our second hypothesis, no significant differences were observed in knee loading between patients with early knee OA and healthy subjects during step-up-and-over. However, most patients with established knee OA presented a different timing of the highest peak medial KCF compared with the other two groups. This difference in the loading pattern observed in patients with established OA needs further analysis.

Interestingly, during step-up-and-over, no significant differences in peak KAM were found between early OA and healthy subjects, or even between established OA and controls, although the high variation in the data indicates larger subject numbers may be necessary to effectively study this task. Nevertheless, patients with established knee OA showed reduced first peak KFM compared to the control group and also to the early knee OA group during the upward propulsive phase (step ascent). This finding is in line with previous studies in stair negotiation [21,22,36,79], in which patients with established knee OA also presented altered movement strategies in the sagittal plane.

### Limitations of this study

These results have to be interpreted in view of some methodological limitations, as inherent to the model used [54]. Firstly, we used a single generic knee model that was scaled to represent the anthropometry of the subjects instead of considering the subject-specific articular geometries, including those of the tibia plateau. Our model does not account for OA induced changes in the articular geometry, such as thickness and mechanical properties of the cartilage, or changes in the muscle and ligament properties. Consequently, the reported differences in KCF and contact pressures only result from altered kinematic and kinetic behavior. Bone deformities, ligament laxity or changes in cartilage induced by joint degeneration were not taken into account and they might produce an effect on contact pressures [80]. However, the effect of having a 2-mm constant cartilage thickness instead of a variable thickness on tibiofemoral contact pressure during gait has been previously assessed and showed limited effect on the observed peak contact pressure (about 4%) [80]. Secondly, although the secondary tibiofemoral kinematics and patellofemoral kinematics were calculated as a function of muscle and ligament forces, and cartilage contact and only knee flexion was tracked in the gait simulation, the method may still present some sensitivity to soft tissue artifacts. Thirdly, although the validation of the model has shown a good agreement between the calculated and experimental kinematics and contact forces in healthy subjects and patients following total knee replacement [54], this validation cannot easily be extended to an OA population. The presence of increased co-contraction, bone deformities or changes in cartilage mechanical properties, and the potential presence of ligament laxity induced by joint degeneration were not evaluated. Therefore, this model might present specific limitations when used in patients with knee OA, especially those known to present increased co-contraction (KL≥2) resulting in an underestimation of the joint loading [81,82]. Compared to previous literature in subjects with instrumented prosthesis [63,64], the magnitude of KCF in healthy subjects and those with OA seen in the present study were higher for both walking and step-up-and-over. Our controls exhibited an averaged peak total resultant KCF of 3.16 BW and 4.94 BW (walking and step-up, respectively), while our patients with knee OA exhibited a peak KCF of 3.91 BW and 4.49 BW, respectively. Reported values in instrumented knee studies range from 2.20 to 2.8 BW for walking and from 2.50 BW to 3.5 BW for stair negotiation [63,64,83–86]. However, KCF measured from instrumented prostheses cannot be expected to be similar to those estimated in healthy and patients with OA, as the surgical procedure involves articular surface replacement, changes in the bone structure, and re-alignment of the mechanical knee axis [87] that dramatically change the gait pattern [88]. In other computational studies [16,23,89], also higher KCFs were observed in healthy and patients with OA. Healthy subjects exhibited a peak total resultant KCF range from 3.00 to 4.35 BW [16,23,89,90], and patients with severe knee OA range from 4.0 to 4.5 BW during walking [16,23,89]. Finally, only females participated in the study and, therefore, no conclusions can be drawn for male patients affected with OA.

## Conclusions

Altered knee joint loading and pressure location during gait were found to be already present in early OA, as confirmed in the elevated medial KCFs and a shift in the center of pressure. Our findings indicate that medial KCF predicted by a novel musculoskeletal simulation routine provides a more helpful metric than the KAM used by previous researchers to identify early knee OA development prior to the onset of radiographic evidences. This reinforces the importance of considering the muscle and ligament forces when assessing knee loading rather than only the external knee adduction moment. Consequently, KCF might be used as feedback signal during gait retraining sessions aiming at controlling knee loading in patients with knee osteoarthritis. Excessive medial KCF seems to be already present in early stages of OA.

As more muscular demanding, step-up-and-over resulted in higher total knee contact force compared to walking in controls, and caused patients to present a large variability in their movement execution, possibly aiming to reduce knee loading. Therefore, step-up-and-over was not the best task to induce higher loading in order to discriminate loading profiles between patients with early knee OA from healthy subjects.

## Supporting information

S1 Fig Averaged knee rotations in the sagittal, frontal and transversal planes during stance phase of gait. The gray shaded area corresponds to the standard deviation of the control group. ^*^ indicates a significant difference between established OA and control groups. ^**#**^ indicates a significant difference between early OA and control group.
(TIF)

S2 Fig Averaged knee rotations in the sagittal, frontal and transversal planes during stance phase of step-up-and-over. The gray shaded area corresponds to the standard deviation of the control group.
(TIF)

S1 Table All peaks of the KCF, KFM, KAM and KRM and rotation angles at the time instant of the first peak MKCF during gait. Peak and minima SS values of the KCF, KFM, KAM and KRM during the stance phase of gait, as well as rotation angles (RAngle in *degrees*). at the time instant of the first peak MKCF, for control (C0), early OA (EA) and established OA (ES) groups.
(DOCX)

S2 Table All peaks of the KCF, KFM, KAM and KRM during step-up-and-over. Peak and SS values of the KCF, KFM and KAM during the stance phase of step-up-and-over for control (C0), early OA (EA) and established OA (ES) groups.
(DOCX)

S3 Table Knee contact forces per subject during gait. First and second peaks of the KCF during gait, and minimum values during midstance (SS).
(DOCX)

S4 Table Knee contact forces per subject during step-up-and-over. First and second peaks of the KCF during step-up-and-over, and minimum values during midstance (SS) of step-up-and-over.
(DOCX)

S5 Table Moments per subject during gait. Peaks of the KAM, KFM and KRM during gait, and minimum value during midstance (SS).
(DOCX)

S6 Table Moments per subject during step-up-and-over. Peaks of the KAM and KFM during step-up-and-over, and minimum value during midstance (SS).
(DOCX)

S7 Table CoP location at anterior-posterior (AP) and lateral-medial (LM) direction. (DOCX)

S8 Table Adduction-abduction angles at the first peak MKCF. (DOCX)
