# Supplementary figures and images for: Medial knee loading is altered in subjects with early osteoarthritis during gait but not during step-up-and-over task
Source: PLoS One. 2017 Nov 8;12(11):e0187583. doi: 10.1371/journal.pone.0187583 (PMC5678707; doi:10.1371/journal.pone.0187583)

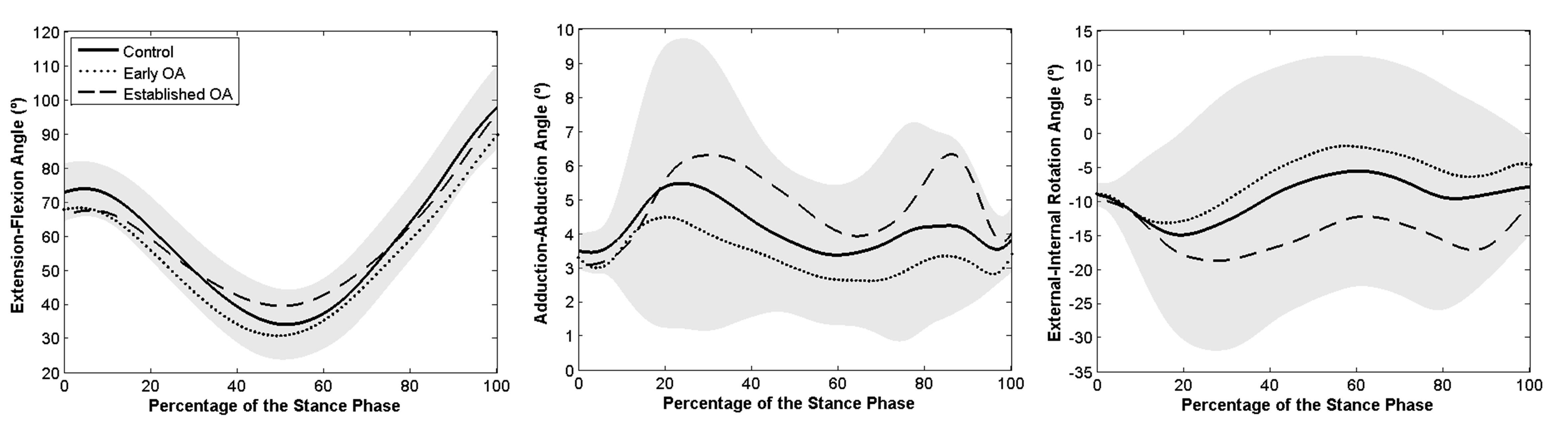

Supplement: S1 Fig — The gray shaded area corresponds to the standard deviation of the control group. * indicates a significant difference between established OA and control groups. # indicates a significant difference between early OA and control group. (TIF) [file pone.0187583.s001.tif]

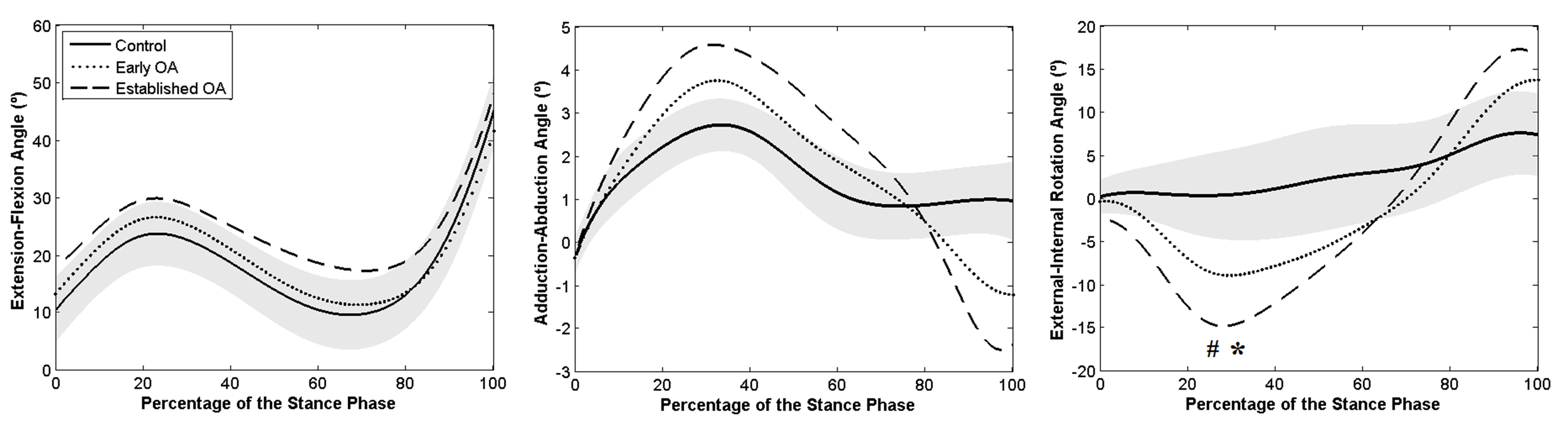

Supplement: S2 Fig — The gray shaded area corresponds to the standard deviation of the control group. (TIF) [file pone.0187583.s002.tif]
