# Supplementary material for: Medial knee loading is altered in subjects with early osteoarthritis during gait but not during step-up-and-over task
Source: PLoS One. 2017 Nov 8;12(11):e0187583. doi: 10.1371/journal.pone.0187583 (PMC5678707; doi:10.1371/journal.pone.0187583)
Supplement: S1 Table — Peak and minima SS values of the KCF, KFM, KAM and KRM during the stance phase of gait, as well as rotation angles (RAngle in degrees). at the time instant of the first peak MKCF, for control (C0), early OA (EA) and established OA (ES) groups. (DOCX) [file pone.0187583.s003.docx]

**S1 Table. All peaks of the KCF, KFM, KAM and KRM and rotation angles** at the **time instant of the first peak MKCF during gait.**

Peak and minima SS values of the KCF, KFM, KAM and KRM during the stance phase of gait, as well as rotation angles (RAngle in *degrees*). at the time instant of the first peak MKCF, for control (C0), early OA (EA) and established OA (ES) groups.

| **Total**  **(80)** | | **Control**  **(34 legs)** | **Early OA**  **(21 legs)** | **Established OA**  **(25 legs)** | ***p*** | ***p***  **(C0**  **vs**  **EA)** | ***p***  **(C0**  **vs**  **ES)** | ***p***  **(EA**  **vs**  **ES)** | ***f*** | ***Power*** |
| --- | --- | --- | --- | --- | --- | --- | --- | --- | --- | --- |
| **P1** | **KFM** | 0.043±0.017 | 0.050±0.019 | 0.048±0.021 | 0.392 | 0.487 | 0.694 | 0.983 | 0.16 | 0.22 |
|  | **KAM** | 0.026±0.005 | 0.026±0.010 | 0.028±0.008 | 0.614 | 0.964 | 0.885 | 0.699 | 0.12 | 0.15 |
|  | **KRM** | 0.003±0.003 | 0.015±0.013 | 0.015±0.014 | **0.001*** | **0.000** | **0.000** | 1.000 | **0.57** | **0.99** |
|  | **TKCF** | 3.16±0.74 | 3.77±0.95 | 3.91±1.23 | **0.008*** | 0.073 | **0.012** | 0.941 | **0.35** | **0.80** |
|  | **MKCF** | 2.17±0.40 | 2.61±0.67 | 2.84±0.89 | **0.001*** | **0.048** | **0.001** | 0.549 | **0.45** | **0.95** |
|  | **LKCF** | 1.18±0.41 | 1.40±0.49 | 1.28±0.56 | 0.265 | 0.275 | 0.841 | 0.760 | 0.18 | 0.29 |
| **P2** | **KFM** | 0.026±0.011 | 0.026±0.008 | 0.030±0.013 | 0.277 | 0.989 | 0.449 | 0.380 | 0.17 | 0.24 |
|  | **KRM** | -0.005±0.003 | -0.010±0.012 | -0.012±0.016 | **0.047** | 0.360 | **0.046** | 0.805 | 0.28 | 0.60 |
|  | **TKCF** | 2.84±0.39 | 3.12±0.40 | 3.28±0.82 | **0.014** | 0.199 | **0.013** | 0.748 | **0.34** | **0.76** |
|  | **MKCF** | 1.80±0.30 | 1.89±0.46 | 2.06±0.55 | 0.084 | 0.869 | 0.078 | 0.437 | 0.26 | 0.50 |
|  | **LKCF** | 1.21±0.27 | 1.48±0.48 | 1.56±0.57 | **0.007** | 0.086 | **0.009** | 0.880 | **0.36** | **0.81** |
| **SS** | **KFM** | -0.007±0.013 | -0.005±0.014 | 0.007±0.017 | **0.001** | 0.984 | **0.002** | **0.014** | **0.42** | **0.92** |
|  | **TKCF** | 1.43±0.20 | 1.58±0.35 | 1.83±0.50 | **0.000** | 0.347 | **0.000** | 0.053 | **0.48** | **0.97** |
|  | **MKCF** | 1.12±0.18 | 1.20±0.28 | 1.40±0.36 | **0.001** | 0.665 | **0.000** | **0.033** | **0.44** | **0.94** |
|  | **LKCF** | 0.32±0.13 | 0.36±0.25 | 0.37±0.26 | 0.570 | 0.844 | 0.679 | 0.996 | 0.11 | 0.12 |
| **RAngle** | | 0.30±5.10 | -7.45±14.00 | -14.61±14.28 | **0.000** | **0.042** | **0.000** | 0.098 | **0.71** | **1.00** |

Statistically significances (*p <* 0.05) are indicated in bold and calculated by *post-hoc* Gabriel calculated by ANOVA. KFM, KAM and KRM are expressed as mean ± SD (BW*Ht), and KCF as (mean ± SD (BW)), where SD is standard deviation. P1 and P2 correspond, respectively, to first and second peak and SS to the minimum value during the single support phase.

Positive values of RAngle indicate internal rotation and negative values indicate external rotation.
