# Supplementary material for: Medial knee loading is altered in subjects with early osteoarthritis during gait but not during step-up-and-over task
Source: PLoS One. 2017 Nov 8;12(11):e0187583. doi: 10.1371/journal.pone.0187583 (PMC5678707; doi:10.1371/journal.pone.0187583)
Supplement: S2 Table — Peak and SS values of the KCF, KFM and KAM during the stance phase of step-up-and-over for control (C0), early OA (EA) and established OA (ES) groups. (DOCX) [file pone.0187583.s004.docx]

**S2 Table. All peaks of the KCF, KFM, KAM and KRM during step-up-and-over.**

Peak and SS values of the KCF, KFM and KAM during the stance phase of step-up-and-over for control (C0), early OA (EA) and established OA (ES) groups.

|  | | **Control**  **(37 legs)** | **Early OA**  **(25 legs)** | **Established OA**  **(24 legs)** | ***p*** | ***p***  **(C0**  **vs**  **EA)** | ***p***  **(C0**  **vs**  **ES)** | ***p***  **(EA**  **vs**  **ES)** | ***f*** | ***Power*** |
| --- | --- | --- | --- | --- | --- | --- | --- | --- | --- | --- |
| **P1** | **KFM** | 0.077±0.025 | 0.077±0.022 | 0.062±0.022 | **0.025*** | 1.000 | **0.038** | 0.060 | 0.29 | 0.65 |
|  | **KAM** | 0.025±0.010 | 0.022±0.010 | 0.022±0.009 | 0.332 | 0.443 | 0.589 | 0.997 | 0.15 | 0.22 |
|  | **TKCF** | 4.94±1.57 | 4.94±1.54 | 4.49±1.47 | 0.467 | 1.000 | 0.588 | 0.654 | 0.13 | 0.17 |
|  | **LKCF** | 2.50±1.32 | 2.63±1.25 | 2.38±1.91 | 0.776 | 0.967 | 0.977 | 0.856 | 0.06 | 0.08 |
| **P2** | **KFM** | 0.101±0.022 | 0.101±0.024 | 0.092±0.019 | 0.214 | 1.000 | 0.278 | 0.373 | 0.19 | 0.31 |
|  | **KAM** | 0.023±0.009 | 0.020±0.007 | 0.022±0.010 | 0.293 | 0.309 | 0.877 | 0.794 | 0.14 | 0.20 |
|  | **TKCF** | 5.64±1.10 | 5.65±1.35 | 5.88±1.29 | 0.719 | 1.000 | 0.835 | 0.873 | 0.08 | 0.10 |
|  | **LKCF** | 2.93±1.00 | 3.08±1.13 | 3.50±1.21 | 0.139 | 0.935 | 0.139 | 0.442 | 0.21 | 0.40 |
| **SS** | **KFM** | 0.028±0.020 | 0.029±0.023 | 0.034±0.024 | 0.609 | 0.999 | 0.707 | 0.832 | 0.12 | 0.14 |
|  | **KAM** | 0.013±0.007 | 0.010±0.008 | 0.012±0.008 | 0.218 | 0.229 | 0.730 | 0.842 | 0.16 | 0.25 |
|  | **TKCF** | 2.55±1.18 | 2.47±1.07 | 3.11±1.26 | 0.102 | 0.990 | 0.186 | 0.151 | 0.23 | 0.45 |
|  | **LKCF** | 0.83±0.59 | 0.86±0.59 | 1.11±0.73 | 0.198 | 0.998 | 0.239 | 0.392 | 0.19 | 0.32 |
| **Highest MKCF** | | 3.16±0.53 | 3.20±0.66 | 3.03±0.79 | 0.589 | 0.989 | 0.809 | 0.691 | 0.10 | 0.12 |

Statistically significances (*p <* 0.05) are indicated in bold and calculated by *post-hoc* Gabriel calculated by ANOVA. KFM, KAM and KRM are expressed as mean ± SD (BW*Ht), and KCF as (mean ± SD (BW)), where SD is standard deviation. P1 and P2 correspond, respectively, to first and second peak and SS to the minimum value during the single support phase.
