# Supplementary material for: Medial knee loading is altered in subjects with early osteoarthritis during gait but not during step-up-and-over task
Source: PLoS One. 2017 Nov 8;12(11):e0187583. doi: 10.1371/journal.pone.0187583 (PMC5678707; doi:10.1371/journal.pone.0187583)
Supplement: S3 Table — First and second peaks of the KCF during gait, and minimum values during midstance (SS). (DOCX) [file pone.0187583.s005.docx]

**S3 Table. Knee contact forces per subject during gait.**

First and second peaks of the KCF during gait, and minimum values during midstance (SS).

| **PATIENT NUMBER** | **TKCF**  **P1** | **TKCF**  **P2** | **MKCF**  **P1** | **MKCF**  **P2** | **LKCF**  **P1** | **LKCF**  **P2** | **TKCF**  **SS** | **MKCF**  **SS** | **LKCF**  **SS** |
| --- | --- | --- | --- | --- | --- | --- | --- | --- | --- |
| 1 | 1.866123 | 3.037541 | 1.484649 | 1.792241 | 0.699340 | 1.382612 | 1.641997 | 1.242603 | 0.365586 |
| 1 | 2.181484 | 2.877649 | 1.832090 | 1.648665 | 0.702600 | 1.361600 | 1.688573 | 1.455618 | 0.181193 |
| 1 | 2.491072 | 3.349903 | 1.848917 | 2.218464 | 0.731134 | 1.206977 | 1.387189 | 1.255335 | 0.154195 |
| 1 | 2.771275 | 2.214871 | 1.795661 | 1.389190 | 1.093612 | 0.885768 | 1.266267 | 0.888908 | 0.414249 |
| 1 | 2.876442 | 2.173790 | 1.852555 | 1.202942 | 1.283156 | 1.210000 | 1.387006 | 0.957881 | 0.395838 |
| 1 | 3.407670 | 3.031999 | 2.312096 | 1.746152 | 1.322701 | 1.429525 | 1.228003 | 0.876439 | 0.382138 |
| 1 | 2.297097 | 2.786833 | 1.536468 | 1.837540 | 0.871188 | 1.063538 | 1.336166 | 1.148712 | 0.197356 |
| 1 | 2.963765 | 2.641580 | 2.190828 | 1.682310 | 0.993946 | 1.065671 | 1.226505 | 0.974976 | 0.244193 |
| 1 | 2.638105 | 2.771057 | 1.870474 | 1.689675 | 0.979587 | 1.191007 | 1.297672 | 1.077138 | 0.217001 |
| 1 | 3.063099 | 3.185044 | 2.134300 | 1.770495 | 1.006893 | 1.552381 | 1.139133 | 0.922695 | 0.222133 |
| 1 | 2.769566 | 3.283484 | 2.133416 | 2.198538 | 0.707353 | 1.241959 | 1.459232 | 1.279361 | 0.161379 |
| 1 | 3.624114 | 2.339170 | 2.354886 | 1.597444 | 1.483305 | 0.973600 | 1.184323 | 0.967462 | 0.209928 |
| 1 | 2.945961 | 2.508454 | 2.022313 | 1.908898 | 1.075659 | 0.678987 | 1.219201 | 1.129457 | 0.071562 |
| 1 | 3.357686 | 2.276920 | 2.362230 | 1.602344 | 1.076567 | 0.855800 | 1.570778 | 1.344672 | 0.255235 |
| 1 | 3.073983 | 2.482608 | 2.147149 | 1.836027 | 0.984096 | 1.053000 | 1.516556 | 1.255515 | 0.272255 |
| 1 | 5.010518 | 3.101968 | 2.814311 | 1.619122 | 2.347224 | 1.832171 | 1.223471 | 0.865128 | 0.391151 |
| 1 | 4.392342 | 3.146293 | 2.677223 | 1.903668 | 1.940307 | 1.475000 | 1.385872 | 1.129985 | 0.278621 |
| 1 | 1.959763 | 2.812450 | 1.570189 | 1.794684 | 0.707937 | 1.146423 | 1.385255 | 1.210981 | 0.131559 |
| 1 | 2.673588 | 2.615038 | 2.075702 | 1.632087 | 0.738806 | 1.080671 | 1.672207 | 1.137327 | 0.438789 |
| 1 | 3.014024 | 2.882480 | 2.263829 | 1.967125 | 1.102011 | 1.033654 | 1.425553 | 1.178386 | 0.273585 |
| 1 | 2.624871 | 2.105125 | 1.778026 | 1.241980 | 0.937700 | 0.942566 | 1.465260 | 1.027985 | 0.467892 |
| 1 | 3.459061 | 2.333952 | 1.961719 | 1.377671 | 1.778783 | 1.050357 | 1.283048 | 0.906377 | 0.386872 |
| 1 | 3.860865 | 3.501361 | 2.513680 | 2.670342 | 1.662853 | 1.316000 | 2.011257 | 1.621742 | 0.461976 |
| 1 | 2.776888 | 2.944633 | 1.759688 | 1.687348 | 1.236801 | 1.388221 | 1.477969 | 1.122310 | 0.394796 |
| 1 | 3.523265 | 2.823610 | 2.455617 | 1.952615 | 1.305124 | 0.961091 | 1.190571 | 0.881774 | 0.322075 |
| 1 | 2.663103 | 2.633335 | 1.736222 | 1.697010 | 1.338510 | 1.083576 | 1.601026 | 1.052345 | 0.577889 |
| 1 | 3.234491 | 2.951968 | 2.471910 | 2.133148 | 0.913950 | 0.967621 | 1.386452 | 1.092458 | 0.305424 |
| 1 | 2.848728 | 3.391264 | 2.256446 | 2.294975 | 0.824989 | 1.255614 | 1.370296 | 1.175806 | 0.184183 |
| 1 | 4.836392 | 3.583683 | 3.233625 | 2.090648 | 1.826135 | 1.727877 | 1.259087 | 0.955022 | 0.323473 |
| 1 | 3.018961 | 2.849861 | 2.044092 | 1.922570 | 1.177689 | 1.041983 | 1.743703 | 1.306718 | 0.493693 |
| 1 | 3.180106 | 2.692720 | 2.300551 | 1.764461 | 1.038854 | 1.040407 | 1.450683 | 1.215942 | 0.266781 |
| 1 | 3.374827 | 2.860507 | 2.421332 | 1.829529 | 1.034075 | 1.249131 | 1.819981 | 1.241317 | 0.639329 |
| 1 | 4.347423 | 3.045179 | 2.791197 | 1.651972 | 1.690493 | 1.740251 | 1.204519 | 0.940411 | 0.275280 |
| 1 | 4.161083 | 3.435858 | 2.696405 | 2.000348 | 1.653918 | 1.612024 | 1.612682 | 1.169371 | 0.482029 |
| 2 | 2.856721 | 2.938401 | 1.974608 | 1.431090 | 1.071339 | 1.938764 | 1.365491 | 1.176000 | 0.216252 |
| 2 | 2.932501 | 2.554506 | 2.139704 | 1.702388 | 1.103345 | 0.975594 | 1.237641 | 1.151901 | 0.094234 |
| 2 | 3.752778 | 3.078943 | 2.420082 | 1.232982 | 1.450528 | 2.235892 | 1.886782 | 1.111969 | 0.648303 |
| 2 | 3.570561 | 3.267845 | 2.290142 | 2.174530 | 1.647328 | 1.263649 | 1.150811 | 0.938857 | 0.239575 |
| 2 | 5.374501 | 3.781457 | 3.925270 | 1.839000 | 1.515819 | 2.389074 | 2.304286 | 2.086000 | 0.248255 |
| 2 | 5.187607 | 2.754376 | 3.281824 | 1.583734 | 2.110674 | 1.519449 | 2.202671 | 1.394000 | 0.785759 |
| 2 | 4.702357 | 3.056070 | 3.345599 | 1.766500 | 1.581267 | 1.556000 | 1.284941 | 0.984079 | 0.315328 |
| 2 | 4.460049 | 3.749503 | 2.755212 | 2.201449 | 1.867907 | 1.761929 | 1.847576 | 1.556805 | 0.306960 |
| 2 | 2.103781 | 2.902037 | 1.617767 | 1.975151 | 0.734462 | 1.133291 | 1.462920 | 1.232836 | 0.179428 |
| 2 | 2.942845 | 2.836107 | 2.008724 | 1.698051 | 1.292364 | 1.242428 | 1.953839 | 1.267437 | 0.723318 |
| 2 | 3.152737 | 2.464131 | 1.755892 | 1.528652 | 1.660419 | 1.022522 | 1.335049 | 0.963220 | 0.407435 |
| 2 | 3.034330 | 2.930956 | 2.192685 | 1.858982 | 1.174139 | 1.451292 | 1.331723 | 1.122683 | 0.215338 |
| 2 | 3.841405 | 2.883682 | 2.664412 | 2.058763 | 1.363802 | 0.917812 | 1.346341 | 1.063223 | 0.311055 |
| 2 | 1.992615 | 3.101204 | 1.854249 | 2.333122 | 0.326101 | 0.909649 | 1.372691 | 1.315080 | 0.000259 |
| 2 | 4.134999 | 3.259674 | 2.651329 | 2.083615 | 1.616742 | 1.334311 | 1.347529 | 0.950887 | 0.375399 |
| 2 | 4.779241 | 2.652238 | 2.568183 | 1.226514 | 2.489328 | 1.837586 | 1.048179 | 0.779544 | 0.271347 |
| 2 | 4.817599 | 3.257553 | 3.164253 | 1.006000 | 1.877157 | 2.535575 | 1.843091 | 0.891654 | 0.953304 |
| 2 | 3.163190 | 3.531447 | 2.168066 | 2.366105 | 1.251139 | 1.332420 | 1.635281 | 1.179040 | 0.518328 |
| 2 | 4.088709 | 3.953280 | 3.886235 | 2.947181 | 0.678994 | 1.254273 | 1.878680 | 1.332421 | 0.028728 |
| 2 | 3.901393 | 3.261986 | 3.047503 | 2.338704 | 1.187836 | 1.066155 | 1.415949 | 1.137020 | 0.301982 |
| 2 | 4.334679 | 3.427164 | 3.151990 | 2.269077 | 1.469263 | 1.309891 | 1.872327 | 1.469559 | 0.453546 |
| 3 | 2.371381 | 1.711561 | 1.748594 | 1.332950 | 0.730179 | 1.006000 | 1.548301 | 1.271796 | 0.280574 |
| 3 | 2.369401 | 2.238438 | 1.610812 | 1.443880 | 1.133910 | 1.148000 | 1.496311 | 1.207255 | 0.301385 |
| 3 | 2.626585 | 2.303534 | 1.854123 | 1.616856 | 0.934056 | 0.819281 | 1.619934 | 1.441813 | 0.208425 |
| 3 | 2.905263 | 2.768230 | 2.110853 | 1.858058 | 0.910838 | 1.012180 | 1.351029 | 1.216193 | 0.145951 |
| 3 | 3.917813 | 3.172512 | 2.592440 | 1.338897 | 1.545565 | 2.060274 | 1.113716 | 0.835342 | 0.290207 |
| 3 | 3.628147 | 3.741734 | 2.180521 | 1.816796 | 1.655430 | 2.139170 | 1.573039 | 0.971342 | 0.606928 |
| 3 | 2.840892 | 3.135660 | 2.535493 | 1.997467 | 0.384228 | 2.177627 | 1.657910 | 1.426018 | 0.042232 |
| 3 | 3.943903 | 3.471617 | 3.216710 | 2.364511 | 0.826939 | 1.272569 | 2.528365 | 2.047082 | 0.254317 |
| 3 | 4.544052 | 4.195800 | 3.335275 | 2.710858 | 1.436376 | 1.643354 | 2.132961 | 1.770620 | 0.382548 |
| 3 | 6.078030 | 4.016837 | 3.386196 | 2.110895 | 2.823642 | 2.665331 | 2.057804 | 1.503815 | 0.469274 |
| 3 | 3.800517 | 3.114810 | 3.154641 | 1.364000 | 0.871750 | 2.100971 | 2.285787 | 1.503000 | 0.499516 |
| 3 | 6.127919 | 2.908796 | 3.916380 | 1.907413 | 2.442876 | 1.291578 | 2.257959 | 1.726595 | 0.543136 |
| 3 | 2.218586 | 2.550037 | 1.518248 | 1.747339 | 0.836001 | 0.884352 | 1.548767 | 1.219007 | 0.342034 |
| 3 | 2.006320 | 2.393022 | 1.497389 | 1.519445 | 0.703802 | 0.964905 | 1.805424 | 1.134359 | 0.587538 |
| 3 | 4.583947 | 4.406402 | 3.021631 | 2.473776 | 1.757638 | 2.275423 | 2.688791 | 2.134622 | 0.583549 |
| 3 | 3.551489 | 4.065952 | 2.028501 | 1.893183 | 1.738251 | 2.578189 | 3.012777 | 1.599706 | 1.338382 |
| 3 | 3.353317 | 2.383501 | 2.302996 | 1.566313 | 1.358568 | 0.888712 | 1.173308 | 0.926190 | 0.241070 |
| 3 | 4.840439 | 4.203567 | 3.656470 | 2.686968 | 1.333016 | 1.811250 | 1.659696 | 1.451934 | 0.231722 |
| 3 | 3.862008 | 3.342235 | 3.005958 | 2.044835 | 0.971694 | 1.567612 | 2.432272 | 1.726699 | 0.667152 |
| 3 | 4.466101 | 2.977271 | 3.508543 | 2.306367 | 1.168295 | 1.296000 | 1.997073 | 1.886008 | 0.113488 |
| 3 | 3.558611 | 3.341237 | 3.148801 | 2.388147 | 0.853878 | 1.065173 | 2.024173 | 1.645305 | 0.153252 |
| 3 | 4.089682 | 5.253090 | 3.508872 | 3.531910 | 0.872603 | 2.051776 | 1.775926 | 1.217407 | 0.216558 |
| 3 | 5.358197 | 3.671237 | 4.299771 | 2.976531 | 1.442888 | 1.262000 | 1.130802 | 0.831393 | 0.303839 |
| 3 | 6.504172 | 3.687935 | 4.809609 | 2.514326 | 1.851674 | 1.906000 | 1.200843 | 0.958834 | 0.227367 |
| 3 | 4.306805 | 2.831751 | 3.186756 | 2.053326 | 1.367815 | 1.151009 | 1.787755 | 1.491880 | 0.334105 |

TKCF, MKCF and LKCF correspond, respectively, to the total knee contact force, medial knee contact force and lateral knee contact force. Values are expressed per unit of body weight (BW).

P1 and P2 correspond, respectively, to first and second peak and SS to the minimum value during the single support phase.
