# Supplementary material for: Medial knee loading is altered in subjects with early osteoarthritis during gait but not during step-up-and-over task
Source: PLoS One. 2017 Nov 8;12(11):e0187583. doi: 10.1371/journal.pone.0187583 (PMC5678707; doi:10.1371/journal.pone.0187583)
Supplement: S4 Table — First and second peaks of the KCF during step-up-and-over, and minimum values during midstance (SS) of step-up-and-over. (DOCX) [file pone.0187583.s006.docx]

**S4 Table. Knee contact forces per subject during step-up-and-over.**

First and second peaks of the KCF during step-up-and-over, and minimum values during midstance (SS) of step-up-and-over.

| **PATIENT NUMBER** | **TKCF**  **P1** | **TKCF**  **P2** | **MKCF**  **P1** | **LKCF**  **P1** | **LKCF**  **P2** | **TKCF**  **SS** | **LKCF**  **SS** |
| --- | --- | --- | --- | --- | --- | --- | --- |
| 1 | 3.317294 | 5.597632 | 2.826629 | 1.527403 | 3.185832 | 1.747964 | 0.779889 |
| 1 | 1.896168 | 3.743313 | 2.955083 | 0.553100 | 1.280000 | 1.620097 | 0.000000 |
| 1 | 3.928184 | 5.421957 | 2.691607 | 1.762822 | 3.097785 | 1.680963 | 0.370736 |
| 1 | 3.783104 | 3.974781 | 2.095413 | 1.849433 | 2.054416 | 2.138875 | 0.858481 |
| 1 | 4.973532 | 5.193311 | 3.365036 | 1.771877 | 2.045414 | 2.427767 | 0.654676 |
| 1 | 3.938448 | 5.290117 | 2.449852 | 2.069376 | 3.010015 | 1.250516 | 0.741104 |
| 1 | 3.178465 | 3.941315 | 2.434898 | 1.197209 | 1.673213 | 1.430928 | 0.179441 |
| 1 | 3.359404 | 4.723754 | 2.943963 | 1.020875 | 1.978404 | 1.582014 | 0.110933 |
| 1 | 3.958195 | 4.270058 | 2.744981 | 1.347121 | 2.173000 | 1.485767 | 0.292515 |
| 1 | 3.216952 | 3.972847 | 2.414640 | 1.402103 | 1.775560 | 2.101549 | 0.613488 |
| 1 | 4.301218 | 5.135260 | 3.097696 | 1.458041 | 2.255114 | 1.510654 | 0.308575 |
| 1 | 3.979412 | 5.665507 | 3.184726 | 1.284924 | 2.810138 | 2.225619 | 0.669265 |
| 1 | 4.664958 | 5.310757 | 3.365867 | 1.702435 | 2.462152 | 2.298641 | 0.515733 |
| 1 | 4.618027 | 4.500117 | 3.695170 | 1.953246 | 1.059194 | 1.692824 | 0.211930 |
| 1 | 6.317211 | 5.433249 | 3.814271 | 3.083928 | 2.378000 | 4.231346 | 0.949283 |
| 1 | 5.438222 | 6.335285 | 3.007209 | 3.168592 | 4.047007 | 3.591419 | 0.777434 |
| 1 | 6.936226 | 7.017400 | 3.319337 | 4.802014 | 3.970289 | 3.876984 | 1.162341 |
| 1 | 6.315567 | 6.828078 | 3.590581 | 3.785690 | 3.876117 | 3.349475 | 0.836981 |
| 1 | 3.850213 | 6.284886 | 2.819822 | 1.961402 | 3.624630 | 1.818592 | 0.982726 |
| 1 | 4.610943 | 4.386480 | 2.414444 | 2.565211 | 2.152444 | 2.399711 | 0.852624 |
| 1 | 4.929204 | 5.719306 | 2.780724 | 2.545622 | 3.173371 | 1.653650 | 0.396342 |
| 1 | 3.401096 | 5.787505 | 3.276640 | 1.872833 | 2.664087 | 1.886369 | 0.955110 |
| 1 | 6.074289 | 6.390211 | 4.005451 | 2.559314 | 2.648673 | 3.291785 | 1.212697 |
| 1 | 4.265844 | 6.045066 | 2.980963 | 2.290319 | 3.250083 | 1.447429 | 0.546663 |
| 1 | 3.690854 | 5.418623 | 2.882792 | 1.765757 | 2.811204 | 2.076081 | 0.795491 |
| 1 | 4.436367 | 5.872176 | 3.049066 | 1.858717 | 2.979292 | 1.719949 | 0.612868 |
| 1 | 4.551266 | 5.077127 | 2.689983 | 2.261371 | 2.586302 | 2.140399 | 1.048037 |
| 1 | 6.973327 | 7.427114 | 4.070560 | 3.081745 | 3.863330 | 3.301114 | 1.215496 |
| 1 | 4.028340 | 6.034130 | 3.353897 | 1.841455 | 2.838613 | 2.094123 | 0.719432 |
| 1 | 4.213301 | 5.274061 | 2.997372 | 1.703140 | 2.445729 | 1.628346 | 0.364827 |
| 1 | 5.396214 | 6.304868 | 3.310664 | 2.256199 | 3.344259 | 3.730799 | 1.426599 |
| 1 | 5.453912 | 6.073570 | 3.129701 | 2.686642 | 3.619179 | 2.778032 | 1.046280 |
| 1 | 7.565861 | 4.394465 | 4.275521 | 4.103934 | 1.803627 | 2.935044 | 0.742688 |
| 1 | 7.891402 | 7.692980 | 3.962656 | 5.098622 | 4.225570 | 5.542976 | 2.096416 |
| 1 | 7.149804 | 6.776987 | 3.348983 | 5.236088 | 5.116879 | 6.260450 | 3.088024 |
| 1 | 8.011241 | 7.686766 | 3.603071 | 5.933372 | 4.904000 | 4.386852 | 1.973232 |
| 1 | 8.359322 | 7.844418 | 4.087862 | 5.084488 | 5.165918 | 3.023111 | 0.730476 |
| 2 | 2.321480 | 3.470000 | 1.987692 | 1.163908 | 1.644559 | 1.454149 | 0.000000 |
| 2 | 4.349364 | 6.814056 | 2.977843 | 1.921570 | 4.162163 | 2.572442 | 1.177707 |
| 2 | 4.053846 | 6.276109 | 3.158853 | 2.108921 | 3.322879 | 1.564400 | 0.634093 |
| 2 | 2.585214 | 3.767019 | 2.411933 | 0.875172 | 1.563200 | 2.077428 | 0.337636 |
| 2 | 4.478412 | 5.041060 | 2.943807 | 2.557907 | 2.382012 | 2.166621 | 0.459058 |
| 2 | 4.452865 | 3.727787 | 3.418410 | 1.185505 | 1.654000 | 1.919614 | 0.000000 |
| 2 | 5.799441 | 4.532011 | 3.301243 | 2.879956 | 2.358424 | 2.088161 | 0.873151 |
| 2 | 3.894388 | 4.458715 | 2.952684 | 1.525740 | 1.662884 | 1.543882 | 0.123745 |
| 2 | 7.124858 | 4.272455 | 3.913601 | 4.320817 | 2.048907 | 2.128105 | 0.280761 |
| 2 | 5.912901 | 6.485578 | 3.783179 | 2.380981 | 3.273950 | 3.242737 | 0.881108 |
| 2 | 3.580135 | 5.706056 | 3.049559 | 1.690861 | 2.882071 | 1.593563 | 0.656970 |
| 2 | 4.719771 | 5.531058 | 2.954678 | 2.471967 | 3.320480 | 3.349349 | 1.405276 |
| 2 | 4.054107 | 4.716018 | 2.336446 | 2.459037 | 2.588626 | 1.965955 | 1.166291 |
| 2 | 3.368892 | 3.434071 | 2.051312 | 1.744348 | 1.539587 | 1.332080 | 0.289937 |
| 2 | 4.366213 | 7.384471 | 3.806808 | 1.924447 | 3.962128 | 2.054865 | 0.936887 |
| 2 | 4.054655 | 6.939720 | 3.585562 | 2.130813 | 3.545556 | 1.525002 | 0.684678 |
| 2 | 4.037539 | 4.474189 | 2.618823 | 1.661292 | 2.181404 | 2.302359 | 0.694852 |
| 2 | 5.128306 | 7.906177 | 3.586877 | 2.306469 | 5.094493 | 1.495550 | 0.551724 |
| 2 | 4.131914 | 5.332496 | 2.799467 | 2.122740 | 2.641030 | 2.395936 | 0.819561 |
| 2 | 5.454461 | 5.670565 | 2.784356 | 4.276981 | 4.241000 | 3.040291 | 1.256108 |
| 2 | 6.785267 | 6.488159 | 4.673446 | 2.779520 | 2.433805 | 4.299073 | 1.282634 |
| 2 | 4.816801 | 6.471020 | 3.157405 | 2.926522 | 3.405417 | 2.934433 | 1.454760 |
| 2 | 6.587449 | 6.497364 | 3.564754 | 5.096220 | 4.057934 | 4.576136 | 1.724496 |
| 2 | 8.995801 | 7.426760 | 4.390612 | 5.767882 | 4.340164 | 1.852492 | 0.912856 |
| 2 | 6.182386 | 6.884744 | 3.313961 | 4.469406 | 5.481589 | 2.976526 | 1.088063 |
| 2 | 7.280000 | 7.182428 | 3.921519 | 3.689000 | 4.190959 | 5.721954 | 2.668532 |
| 3 | 6.506443 | 9.077757 | 5.993853 | 3.687209 | 3.886126 | 2.204901 | 0.809215 |
| 3 | 1.924812 | 4.171939 | 2.408651 | 0.744491 | 2.142000 | 1.426628 | 0.024886 |
| 3 | 2.419525 | 4.110279 | 2.860361 | 0.549340 | 1.378509 | 1.787612 | 0.049923 |
| 3 | 3.230111 | 4.151042 | 2.458547 | 1.234107 | 1.894000 | 2.391184 | 0.560965 |
| 3 | 2.725949 | 4.102330 | 2.039934 | 1.500634 | 2.263807 | 2.296269 | 0.994848 |
| 3 | 4.565853 | 4.807531 | 2.617342 | 2.587610 | 3.108948 | 3.176813 | 1.253659 |
| 3 | 5.317193 | 5.428043 | 3.905497 | 2.228556 | 2.357912 | 3.757905 | 0.413595 |
| 3 | 3.042056 | 5.929869 | 3.070486 | 1.135584 | 3.179946 | 1.730594 | 0.511721 |
| 3 | 4.251109 | 6.784621 | 2.635243 | 2.217686 | 4.864793 | 2.657039 | 0.686870 |
| 3 | 6.169299 | 5.805899 | 3.420161 | 2.838681 | 2.793972 | 5.471388 | 2.408509 |
| 3 | 3.529096 | 4.896501 | 3.050876 | 1.317440 | 1.950981 | 1.795267 | 0.484375 |
| 3 | 5.360140 | 6.488589 | 3.161098 | 2.375481 | 3.801513 | 2.208234 | 1.044440 |
| 3 | 4.476798 | 4.700934 | 2.512021 | 2.152074 | 2.477000 | 3.821129 | 1.504132 |
| 3 | 1.915356 | 4.757231 | 2.073682 | 1.234506 | 2.827086 | 1.211309 | 0.382735 |
| 3 | 3.579418 | 6.911812 | 3.134929 | 1.627619 | 3.951028 | 2.772105 | 1.049255 |
| 3 | 3.774424 | 5.608795 | 2.951416 | 1.290754 | 2.849343 | 2.191778 | 0.629330 |
| 3 | 6.131163 | 5.857526 | 2.820429 | 4.197958 | 3.848715 | 4.374597 | 1.900674 |
| 3 | 3.829409 | 5.526934 | 2.578847 | 1.621382 | 4.148075 | 2.946072 | 0.768054 |
| 3 | 5.429657 | 6.399203 | 2.812661 | 4.267048 | 4.530173 | 4.396366 | 1.838300 |
| 3 | 5.676651 | 6.177778 | 3.212300 | 3.791521 | 4.980480 | 2.768220 | 0.728211 |
| 3 | 5.464503 | 6.163799 | 3.537953 | 3.050648 | 3.592565 | 4.290588 | 1.537094 |
| 3 | 5.473416 | 6.531856 | 2.646902 | 3.693789 | 4.646218 | 4.173332 | 1.798863 |
| 3 | 4.374091 | 7.217480 | 3.203536 | 2.410141 | 4.739437 | 3.343787 | 1.551969 |
| 3 | 6.157000 | 8.117764 | 4.069994 | 2.850625 | 5.091879 | 5.577030 | 2.294513 |
| 3 | 7.050891 | 7.367864 | 2.548852 | 4.896048 | 6.078046 | 5.022851 | 2.628542 |

TKCF, MKCF and LKCF correspond, respectively, to the total knee contact force, medial knee contact force and lateral knee contact force. Values are expressed per unit of body weight (BW).

P1 and P2 correspond, respectively, to first and second peak and SS to the minimum value during the single support phase.
