# Supplementary material for: Medial knee loading is altered in subjects with early osteoarthritis during gait but not during step-up-and-over task
Source: PLoS One. 2017 Nov 8;12(11):e0187583. doi: 10.1371/journal.pone.0187583 (PMC5678707; doi:10.1371/journal.pone.0187583)
Supplement: S5 Table — Peaks of the KAM, KFM and KRM during gait, and minimum value during midstance (SS). (DOCX) [file pone.0187583.s007.docx]

**S5 Table. Moments per subject during gait.**

Peaks of the KAM, KFM and KRM during gait, and minimum value during midstance (SS).

| **PATIENT NUMBER** | **KAM**  **P1** | **KRM**  **P1** | **KRM**  **P2** | **KFM**  **P1** | **KFM**  **P2** | **KFM**  **SS** |
| --- | --- | --- | --- | --- | --- | --- |
| 1 | 0.020888 | -0.000269 | -0.006253 | 0.014856 | 0.008135 | -0.019898 |
| 1 | 0.026643 | 0.009394 | -0.010555 | 0.012156 | 0.015667 | -0.021948 |
| 1 | 0.025777 | -0.000356 | -0.011585 | 0.030073 | 0.012712 | -0.023867 |
| 1 | 0.017890 | 0.000031 | -0.007258 | 0.037628 | 0.014901 | -0.007761 |
| 1 | 0.019872 | 0.003371 | -0.002104 | 0.037417 | 0.024255 | 0.001606 |
| 1 | 0.032474 | -0.001741 | -0.005684 | 0.039010 | 0.025529 | -0.025513 |
| 1 | 0.020615 | -0.000512 | -0.004671 | 0.025313 | 0.019313 | -0.005018 |
| 1 | 0.030703 | 0.002233 | -0.005414 | 0.037760 | 0.027919 | -0.013046 |
| 1 | 0.021979 | 0.001424 | -0.006652 | 0.033038 | 0.015636 | -0.020484 |
| 1 | 0.025316 | 0.003362 | -0.008857 | 0.036172 | 0.028843 | -0.021567 |
| 1 | 0.031458 | 0.000834 | -0.008684 | 0.019772 | 0.024784 | -0.025517 |
| 1 | 0.025471 | 0.004038 | -0.007787 | 0.063006 | 0.029025 | -0.002513 |
| 1 | 0.030644 | -0.000645 | -0.009264 | 0.043096 | 0.008746 | -0.005721 |
| 1 | 0.029321 | 0.002644 | -0.005308 | 0.048752 | 0.021662 | -0.001349 |
| 1 | 0.030457 | 0.005321 | -0.004000 | 0.047921 | 0.031698 | 0.007637 |
| 1 | 0.019181 | 0.003712 | -0.007737 | 0.081551 | 0.047223 | 0.006901 |
| 1 | 0.027129 | 0.010038 | -0.002750 | 0.073082 | 0.039739 | 0.003877 |
| 1 | 0.022549 | 0.001451 | -0.001935 | 0.017591 | 0.013601 | -0.015305 |
| 1 | 0.023393 | 0.013323 | -0.007739 | 0.028171 | 0.025714 | -0.004296 |
| 1 | 0.031448 | 0.001746 | -0.005923 | 0.041750 | 0.022872 | -0.013921 |
| 1 | 0.019873 | -0.000194 | -0.001182 | 0.032507 | 0.023079 | 0.005012 |
| 1 | 0.017908 | 0.001814 | 0.001215 | 0.046255 | 0.034353 | 0.002255 |
| 1 | 0.031563 | -0.003341 | -0.007946 | 0.052117 | 0.035649 | -0.015045 |
| 1 | 0.018073 | 0.002147 | 0.000044 | 0.042482 | 0.043082 | 0.012386 |
| 1 | 0.032675 | 0.002787 | -0.001583 | 0.050159 | 0.029804 | -0.004367 |
| 1 | 0.020397 | 0.001414 | -0.002221 | 0.032765 | 0.014380 | -0.014623 |
| 1 | 0.033764 | 0.007168 | -0.004922 | 0.043591 | 0.020773 | -0.015707 |
| 1 | 0.032879 | 0.002720 | -0.005109 | 0.034421 | 0.024885 | -0.025170 |
| 1 | 0.037163 | 0.006729 | -0.005871 | 0.085158 | 0.037396 | 0.005139 |
| 1 | 0.023735 | 0.002342 | -0.003600 | 0.040129 | 0.020491 | -0.013071 |
| 1 | 0.029901 | -0.001436 | -0.003272 | 0.043393 | 0.023950 | 0.005475 |
| 1 | 0.029249 | 0.001668 | -0.001567 | 0.053594 | 0.040819 | 0.020848 |
| 1 | 0.028279 | 0.003342 | -0.005697 | 0.064678 | 0.045607 | 0.003803 |
| 1 | 0.030152 | 0.001093 | -0.003124 | 0.064758 | 0.043719 | 0.011652 |
| 2 | 0.023822 | 0.002709 | -0.005421 | 0.039365 | 0.020297 | -0.018881 |
| 2 | 0.026593 | 0.002004 | -0.006807 | 0.037012 | 0.014822 | -0.015321 |
| 2 | 0.009853 | 0.030976 | -0.025694 | 0.041886 | 0.019068 | -0.012228 |
| 2 | 0.023610 | 0.001537 | -0.007803 | 0.059153 | 0.028378 | -0.003927 |
| 2 | 0.043568 | 0.028411 | -0.039499 | 0.057355 | 0.038188 | 0.021612 |
| 2 | 0.017483 | 0.039835 | -0.020559 | 0.070365 | 0.030220 | -0.004610 |
| 2 | 0.041220 | 0.013881 | -0.014754 | 0.075806 | 0.034181 | -0.004572 |
| 2 | 0.027947 | 0.007031 | -0.010851 | 0.078112 | 0.026876 | -0.002478 |
| 2 | 0.020754 | 0.005846 | -0.000878 | 0.020254 | 0.008846 | -0.018543 |
| 2 | 0.021233 | 0.002592 | 0.000742 | 0.048067 | 0.030451 | 0.025847 |
| 2 | 0.016321 | 0.004208 | -0.000721 | 0.038642 | 0.026196 | -0.008741 |
| 2 | 0.027459 | 0.008943 | -0.003281 | 0.042895 | 0.024654 | -0.018323 |
| 2 | 0.029351 | 0.010835 | -0.001253 | 0.054991 | 0.023258 | -0.012170 |
| 2 | 0.028565 | 0.008913 | -0.007414 | 0.016332 | 0.015592 | -0.022744 |
| 2 | 0.027275 | 0.014800 | 0.000190 | 0.059051 | 0.024758 | -0.007231 |
| 2 | 0.019906 | 0.006083 | -0.001716 | 0.079239 | 0.037967 | -0.002445 |
| 2 | -0.001711 | 0.035006 | -0.016289 | 0.029341 | 0.019849 | -0.007473 |
| 2 | 0.022325 | 0.011505 | -0.005323 | 0.047805 | 0.023168 | -0.014542 |
| 2 | 0.036646 | 0.041349 | -0.033841 | 0.018186 | 0.021370 | -0.009783 |
| 2 | 0.036487 | 0.019893 | -0.003559 | 0.054259 | 0.029126 | -0.003111 |
| 2 | 0.037097 | 0.017728 | 0.002060 | 0.071704 | 0.038573 | 0.024866 |
| 3 | 0.020617 | 0.002868 | -0.005709 | 0.030768 | 0.018234 | 0.013540 |
| 3 | 0.018479 | 0.004236 | -0.003159 | 0.024263 | 0.027602 | 0.006846 |
| 3 | 0.024120 | -0.000454 | -0.005963 | 0.026329 | 0.016352 | -0.013661 |
| 3 | 0.028808 | 0.001966 | -0.009499 | 0.039849 | 0.019153 | -0.015624 |
| 3 | 0.028624 | 0.003382 | -0.002590 | 0.068967 | 0.022554 | -0.017887 |
| 3 | 0.012068 | 0.023333 | -0.040016 | 0.051372 | 0.020674 | -0.010801 |
| 3 | 0.028209 | 0.015174 | -0.016142 | 0.026737 | 0.048750 | 0.010147 |
| 3 | 0.027712 | 0.025401 | -0.021643 | 0.034498 | 0.021740 | 0.012595 |
| 3 | 0.034046 | 0.005314 | -0.004477 | 0.066148 | 0.045539 | 0.025222 |
| 3 | 0.030630 | 0.040586 | -0.037211 | 0.067079 | 0.027453 | -0.001581 |
| 3 | 0.033404 | 0.032707 | -0.033527 | 0.044239 | 0.030631 | 0.016508 |
| 3 | 0.032919 | 0.011900 | 0.030130 | 0.092486 | 0.030210 | 0.032530 |
| 3 | 0.018357 | 0.007849 | 0.002362 | 0.025590 | 0.016141 | -0.012473 |
| 3 | 0.016152 | 0.005047 | 0.000944 | 0.016780 | 0.014222 | -0.010511 |
| 3 | 0.031361 | 0.004168 | -0.005679 | 0.070804 | 0.070909 | 0.040972 |
| 3 | 0.017075 | 0.002218 | -0.006015 | 0.049758 | 0.044813 | 0.028283 |
| 3 | 0.030026 | 0.003118 | -0.001700 | 0.046374 | 0.029688 | 0.001563 |
| 3 | 0.044681 | 0.024835 | -0.001992 | 0.076640 | 0.032302 | 0.007267 |
| 3 | 0.024609 | 0.018896 | -0.016481 | 0.034329 | 0.040418 | 0.027403 |
| 3 | 0.028131 | 0.028915 | -0.012110 | 0.039327 | 0.031321 | 0.010618 |
| 3 | 0.042434 | 0.004860 | -0.005895 | 0.046909 | 0.031562 | 0.018255 |
| 3 | 0.024120 | 0.038970 | -0.032344 | 0.025930 | 0.027882 | 0.002209 |
| 3 | 0.025239 | 0.038100 | -0.035732 | 0.037714 | 0.020706 | -0.018524 |
| 3 | 0.032672 | 0.033375 | -0.033392 | 0.081129 | 0.027081 | -0.000589 |
| 3 | 0.040056 | 0.003788 | -0.008716 | 0.067818 | 0.037977 | 0.019926 |

KFM, KAM and KRM correspond, respectively, to the knee flexion moment, knee adduction moment and knee rotation moment. Values are expressed per unit of body weight times height (BW*Ht).

P1 and P2 correspond, respectively, to first and second peak and SS to the minimum value during the single support phase.
