# Supplementary material for: Medial knee loading is altered in subjects with early osteoarthritis during gait but not during step-up-and-over task
Source: PLoS One. 2017 Nov 8;12(11):e0187583. doi: 10.1371/journal.pone.0187583 (PMC5678707; doi:10.1371/journal.pone.0187583)
Supplement: S6 Table — Peaks of the KAM and KFM during step-up-and-over, and minimum value during midstance (SS). (DOCX) [file pone.0187583.s008.docx]

**S6 Table. Moments per subject during step-up-and-over.**

Peaks of the KAM and KFM during step-up-and-over, and minimum value during midstance (SS).

| **PATIENT NUMBER** | **KAM**  **P1** | **KAM**  **P2** | **KFM**  **P1** | **KFM**  **P2** | **KAM**  **SS** | **KFM**  **SS** |
| --- | --- | --- | --- | --- | --- | --- |
| 1 | 0.008583 | 0.008780 | 0.042045 | 0.086422 | 0.007010 | 0.015196 |
| 1 | 0.030360 | 0.029311 | 0.019960 | 0.070240 | 0.021898 | 0.001023 |
| 1 | 0.014748 | 0.012476 | 0.072718 | 0.120190 | -0.002058 | 0.011062 |
| 1 | 0.016256 | 0.012121 | 0.064688 | 0.085348 | 0.009767 | 0.033214 |
| 1 | 0.029107 | 0.024152 | 0.077258 | 0.092238 | 0.019558 | 0.037354 |
| 1 | 0.007408 | 0.006320 | 0.056569 | 0.086441 | 0.003418 | 0.003738 |
| 1 | 0.025233 | 0.021194 | 0.050277 | 0.080791 | 0.013448 | 0.007113 |
| 1 | 0.028310 | 0.029247 | 0.060065 | 0.101892 | 0.020461 | 0.013054 |
| 1 | 0.020080 | 0.014490 | 0.052136 | 0.075359 | 0.012023 | 0.004219 |
| 1 | 0.018325 | 0.021259 | 0.050615 | 0.082908 | 0.017180 | 0.030038 |
| 1 | 0.023870 | 0.016790 | 0.063317 | 0.071396 | 0.012378 | 0.008272 |
| 1 | 0.023145 | 0.026156 | 0.053224 | 0.084167 | 0.011108 | 0.027586 |
| 1 | 0.022932 | 0.020854 | 0.059586 | 0.071133 | 0.016873 | 0.029365 |
| 1 | 0.030544 | 0.032369 | 0.044924 | 0.050890 | 0.018835 | 0.001292 |
| 1 | 0.048704 | 0.032859 | 0.076179 | 0.080054 | 0.029683 | 0.043188 |
| 1 | 0.041801 | 0.035300 | 0.063974 | 0.086709 | 0.024758 | 0.032078 |
| 1 | 0.045428 | 0.035037 | 0.090663 | 0.117593 | 0.017691 | 0.040922 |
| 1 | 0.033192 | 0.032327 | 0.078334 | 0.100937 | 0.020378 | 0.031268 |
| 1 | 0.012576 | 0.007444 | 0.065249 | 0.123093 | -0.002890 | 0.023528 |
| 1 | 0.012566 | 0.014230 | 0.074956 | 0.080862 | 0.010930 | 0.029460 |
| 1 | 0.013024 | 0.008678 | 0.086772 | 0.120185 | 0.000992 | 0.005456 |
| 1 | 0.019695 | 0.019275 | 0.054925 | 0.101155 | 0.008733 | 0.017355 |
| 1 | 0.035836 | 0.031445 | 0.108455 | 0.126748 | 0.018623 | 0.050744 |
| 1 | 0.012548 | 0.015685 | 0.077175 | 0.122406 | 0.004044 | 0.008792 |
| 1 | 0.016141 | 0.019978 | 0.064296 | 0.113969 | 0.009786 | 0.027881 |
| 1 | 0.020975 | 0.019391 | 0.100085 | 0.121543 | 0.009773 | 0.022326 |
| 1 | 0.017698 | 0.027462 | 0.086040 | 0.125008 | 0.006830 | 0.029550 |
| 1 | 0.026460 | 0.030478 | 0.103452 | 0.117933 | 0.019631 | 0.053914 |
| 1 | 0.021737 | 0.026212 | 0.076350 | 0.124848 | 0.015185 | 0.030461 |
| 1 | 0.025727 | 0.023907 | 0.078062 | 0.109992 | 0.014969 | 0.014409 |
| 1 | 0.030488 | 0.026174 | 0.101208 | 0.116273 | 0.016469 | 0.060000 |
| 1 | 0.024946 | 0.022228 | 0.104847 | 0.099393 | 0.016712 | 0.046910 |
| 1 | 0.028892 | 0.027153 | 0.110230 | 0.088598 | 0.013328 | 0.017237 |
| 1 | 0.039385 | 0.035700 | 0.118062 | 0.117790 | 0.022359 | 0.063959 |
| 1 | 0.035856 | 0.038243 | 0.113972 | 0.127937 | 0.021218 | 0.087598 |
| 1 | 0.033376 | 0.024045 | 0.123948 | 0.142628 | 0.006250 | 0.059584 |
| 1 | 0.031433 | 0.031711 | 0.120041 | 0.128742 | 0.011061 | 0.026751 |
| 2 | 0.024931 | 0.012739 | 0.040335 | 0.071982 | 0.010464 | -0.000749 |
| 2 | 0.011981 | 0.019935 | 0.065158 | 0.099771 | 0.003386 | 0.036373 |
| 2 | 0.010495 | 0.014619 | 0.060986 | 0.096718 | 0.003333 | 0.003055 |
| 2 | 0.026318 | 0.020469 | 0.044076 | 0.077202 | 0.015979 | 0.030153 |
| 2 | 0.019616 | 0.019040 | 0.058131 | 0.055450 | 0.018680 | 0.023240 |
| 2 | 0.032130 | 0.027411 | 0.054515 | 0.072477 | 0.025379 | 0.017370 |
| 2 | 0.014438 | 0.012538 | 0.084720 | 0.074926 | 0.005970 | 0.026870 |
| 2 | 0.024287 | 0.025610 | 0.059150 | 0.083514 | 0.016952 | 0.012765 |
| 2 | 0.054120 | 0.028988 | 0.090366 | 0.075171 | 0.020941 | 0.001415 |
| 2 | 0.034006 | 0.023763 | 0.077973 | 0.092557 | 0.016828 | 0.044489 |
| 2 | 0.013929 | 0.017504 | 0.055503 | 0.114084 | -0.000426 | -0.007563 |
| 2 | 0.020386 | 0.020378 | 0.095179 | 0.102675 | 0.015290 | 0.061518 |
| 2 | 0.013981 | 0.009197 | 0.066219 | 0.091009 | -0.000162 | 0.023343 |
| 2 | 0.020256 | 0.016078 | 0.059320 | 0.071327 | 0.007366 | 0.004674 |
| 2 | 0.024418 | 0.017474 | 0.073567 | 0.113763 | 0.005633 | 0.028719 |
| 2 | 0.013210 | 0.021667 | 0.070221 | 0.128438 | 0.004315 | 0.012153 |
| 2 | 0.026769 | 0.025024 | 0.072911 | 0.094965 | 0.018128 | 0.031552 |
| 2 | 0.017447 | 0.022813 | 0.076813 | 0.127618 | 0.004936 | 0.004496 |
| 2 | 0.022683 | 0.018852 | 0.073504 | 0.108646 | 0.013356 | 0.036959 |
| 2 | 0.013792 | 0.014197 | 0.082986 | 0.099336 | 0.002484 | 0.035288 |
| 2 | 0.039684 | 0.043054 | 0.110571 | 0.134642 | 0.024712 | 0.072421 |
| 2 | 0.011377 | 0.016484 | 0.079598 | 0.123460 | -0.004983 | 0.045733 |
| 2 | 0.009416 | 0.005548 | 0.114719 | 0.108241 | 0.005176 | 0.058709 |
| 2 | 0.020347 | 0.017124 | 0.129824 | 0.145438 | 0.005347 | 0.025754 |
| 2 | 0.018090 | 0.022582 | 0.092569 | 0.143580 | 0.008935 | 0.042175 |
| 2 | 0.025654 | 0.020319 | 0.113824 | 0.123748 | 0.011832 | 0.084771 |
| 3 | 0.019103 | 0.013862 | 0.082429 | 0.087005 | -0.003484 | 0.036345 |
| 3 | 0.020058 | 0.019213 | 0.015069 | 0.056427 | 0.018020 | -0.000563 |
| 3 | 0.026757 | 0.026380 | 0.038469 | 0.075626 | 0.022970 | 0.007216 |
| 3 | 0.023497 | 0.016650 | 0.048910 | 0.070385 | 0.017630 | 0.030636 |
| 3 | 0.009701 | 0.006650 | 0.040188 | 0.064014 | 0.001462 | 0.034076 |
| 3 | 0.010955 | 0.011003 | 0.056502 | 0.060429 | 0.006637 | 0.034363 |
| 3 | 0.047461 | 0.037382 | 0.063765 | 0.076794 | 0.035393 | 0.030939 |
| 3 | 0.019561 | 0.011803 | 0.047561 | 0.104681 | 0.010930 | 0.024538 |
| 3 | 0.019889 | 0.027479 | 0.041685 | 0.097103 | 0.010209 | 0.011967 |
| 3 | 0.036082 | 0.026780 | 0.079909 | 0.085869 | 0.023269 | 0.069288 |
| 3 | 0.020563 | 0.029812 | 0.049491 | 0.095698 | 0.011806 | -0.028878 |
| 3 | 0.015684 | 0.010154 | 0.075576 | 0.103515 | 0.007330 | 0.030807 |
| 3 | 0.022947 | 0.018512 | 0.076236 | 0.079165 | 0.012973 | 0.059961 |
| 3 | 0.006494 | 0.005137 | 0.018659 | 0.089447 | 0.001404 | -0.004302 |
| 3 | 0.021997 | 0.017648 | 0.062024 | 0.107143 | 0.013808 | 0.044204 |
| 3 | 0.031121 | 0.017281 | 0.060878 | 0.107503 | 0.010286 | 0.030475 |
| 3 | 0.012164 | 0.013063 | 0.093726 | 0.089062 | 0.008282 | 0.048223 |
| 3 | 0.019528 | 0.027019 | 0.034394 | 0.073247 | 0.014025 | 0.018418 |
| 3 | 0.031953 | 0.033323 | 0.071323 | 0.101398 | 0.014310 | 0.050994 |
| 3 | 0.028727 | 0.043332 | 0.073122 | 0.107054 | 0.004359 | 0.026371 |
| 3 | 0.021313 | 0.029831 | 0.075536 | 0.094450 | 0.011781 | 0.054028 |
| 3 | 0.022223 | 0.030021 | 0.072253 | 0.113040 | 0.006384 | 0.044457 |
| 3 | 0.012880 | 0.014810 | 0.071688 | 0.121518 | 0.006227 | 0.054555 |
| 3 | 0.028985 | 0.029126 | 0.091311 | 0.127045 | 0.016361 | 0.075601 |
| 3 | 0.025307 | 0.027643 | 0.097157 | 0.115781 | 0.007820 | 0.060969 |

KFM, and KAM correspond, respectively, to the knee flexion moment, and knee adduction moment. Values are expressed per unit of body weight times height (BW*Ht).

P1 and P2 correspond, respectively, to first and second peak and SS to the minimum value during the single support phase.
