# Supplementary material for: Medial knee loading is altered in subjects with early osteoarthritis during gait but not during step-up-and-over task
Source: PLoS One. 2017 Nov 8;12(11):e0187583. doi: 10.1371/journal.pone.0187583 (PMC5678707; doi:10.1371/journal.pone.0187583)
Supplement: S7 Table — (DOCX) [file pone.0187583.s009.docx]

**S7 Table. CoP location at anterior-posterior (AP) and lateral-medial (LM) direction.**

| **PATIENT NUMBER** | **AP** | **LM** |
| --- | --- | --- |
| 1 | 0.860970 | -21.984288 |
| 1 | -2.460737 | -21.738607 |
| 1 | -1.242783 | -21.373691 |
| 1 | -1.962611 | -23.065320 |
| 1 | -3.224630 | -24.038800 |
| 1 | -1.940842 | -24.622023 |
| 1 | -2.705010 | -21.420763 |
| 1 | -2.582353 | -21.455798 |
| 1 | -1.834326 | -22.215302 |
| 1 | -2.809913 | -21.918236 |
| 1 | 0.799439 | -23.339047 |
| 1 | -5.164322 | -22.070905 |
| 1 | -3.879575 | -22.193419 |
| 1 | -2.798023 | -22.032225 |
| 1 | -3.437994 | -22.827312 |
| 1 | -3.997503 | -23.073842 |
| 1 | -4.243891 | -21.592205 |
| 1 | -0.257861 | -21.626875 |
| 1 | -5.669491 | -22.666423 |
| 1 | -0.381550 | -22.277753 |
| 1 | 1.291725 | -24.096393 |
| 1 | 0.436184 | -22.188065 |
| 1 | 1.970465 | -24.078291 |
| 1 | -1.187682 | -24.348766 |
| 1 | -2.762058 | -21.618501 |
| 1 | -1.176377 | -21.781986 |
| 1 | -3.595462 | -23.427093 |
| 1 | -1.042316 | -23.949179 |
| 1 | -2.823661 | -21.182137 |
| 1 | -1.083317 | -21.563505 |
| 1 | 0.323970 | -20.473532 |
| 1 | -3.040742 | -20.702865 |
| 1 | -4.951387 | -22.384853 |
| 1 | -3.981215 | -22.473557 |
| 2 | -0.301806 | -22.856852 |
| 2 | -1.792840 | -19.287474 |
| 2 | -4.668588 | -24.042138 |
| 2 | -4.001588 | -22.844992 |
| 2 | -5.643090 | -19.240450 |
| 2 | -5.165192 | -26.377200 |
| 2 | -5.069640 | -24.895828 |
| 2 | -5.798865 | -21.570602 |
| 2 | -0.458491 | -24.188683 |
| 2 | -1.959729 | -24.123085 |
| 2 | -0.625778 | -21.539838 |
| 2 | -0.541179 | -24.227512 |
| 2 | -1.952580 | -20.152651 |
| 2 | -2.551752 | -21.092029 |
| 2 | -4.688674 | -23.765772 |
| 2 | -2.975607 | -24.418468 |
| 2 | -5.388989 | -21.155380 |
| 2 | -3.998539 | -24.644879 |
| 2 | -5.086081 | -23.370727 |
| 2 | -3.277545 | -22.277000 |
| 2 | -4.186596 | -22.524138 |
| 3 | -4.547149 | -21.861029 |
| 3 | -1.736801 | -17.940827 |
| 3 | 1.179595 | -20.167417 |
| 3 | -2.298350 | -22.915078 |
| 3 | -3.290861 | -22.022360 |
| 3 | -4.190176 | -26.648037 |
| 3 | -4.205597 | -25.969652 |
| 3 | -4.508340 | -23.050382 |
| 3 | -5.440908 | -20.492197 |
| 3 | -4.139335 | -20.633319 |
| 3 | -5.117827 | -22.202622 |
| 3 | -5.927892 | -17.224081 |
| 3 | -3.796446 | -20.798190 |
| 3 | -1.005630 | -21.333171 |
| 3 | -6.404642 | -17.319969 |
| 3 | -3.040262 | -17.965755 |
| 3 | -2.256751 | -23.382749 |
| 3 | -4.222986 | -22.355332 |
| 3 | -4.848194 | -18.284852 |
| 3 | -4.692980 | -21.372022 |
| 3 | -4.736748 | -20.850170 |
| 3 | -4.791102 | -22.632815 |
| 3 | -4.829244 | -20.083080 |
| 3 | -4.560152 | -21.083380 |
| 3 | -4.228799 | -21.170835 |

These values are normalized to the tibia scale factor and expressed in *mm*.
