# Supplementary material for: Medial knee loading is altered in subjects with early osteoarthritis during gait but not during step-up-and-over task
Source: PLoS One. 2017 Nov 8;12(11):e0187583. doi: 10.1371/journal.pone.0187583 (PMC5678707; doi:10.1371/journal.pone.0187583)
Supplement: S8 Table — (DOCX) [file pone.0187583.s010.docx]

**S8 Table. Adduction-abduction angles at the first peak MKCF.**

| **PATIENT NUMBER** | **Adduction-abduction angles (º)** |
| --- | --- |
| 1 | 2.180250 |
| 1 | -10.526200 |
| 1 | 1.898000 |
| 1 | 2.264333 |
| 1 | -2.020750 |
| 1 | 4.458000 |
| 1 | 0.324000 |
| 1 | 1.206667 |
| 1 | 2.070000 |
| 1 | -1.727000 |
| 1 | -0.339000 |
| 1 | -1.350800 |
| 1 | 2.459500 |
| 1 | -1.532000 |
| 1 | -8.146000 |
| 1 | -0.259000 |
| 1 | -4.559667 |
| 1 | -0.043750 |
| 1 | -17.006000 |
| 1 | 2.619250 |
| 1 | 4.557250 |
| 1 | 5.746000 |
| 1 | 10.030500 |
| 1 | 3.520333 |
| 1 | 0.717429 |
| 1 | 2.728667 |
| 1 | -6.975000 |
| 1 | -0.098143 |
| 1 | 2.004333 |
| 1 | 1.666333 |
| 1 | 7.835200 |
| 1 | 2.460857 |
| 1 | 0.186400 |
| 1 | 3.821667 |
| 2 | 1.378333 |
| 2 | 0.421750 |
| 2 | -30.627000 |
| 2 | 4.509333 |
| 2 | -31.894000 |
| 2 | -26.788500 |
| 2 | -6.382333 |
| 2 | -4.932750 |
| 2 | -1.365000 |
| 2 | 5.467000 |
| 2 | 2.238800 |
| 2 | 3.852000 |
| 2 | 3.078600 |
| 2 | -6.306500 |
| 2 | -4.830600 |
| 2 | 4.297250 |
| 2 | -29.106000 |
| 2 | -0.295800 |
| 2 | -36.653500 |
| 2 | -2.251500 |
| 2 | -0.205500 |
| 3 | -4.911500 |
| 3 | -4.105800 |
| 3 | 2.761800 |
| 3 | -0.274500 |
| 3 | 0.583000 |
| 3 | -22.385000 |
| 3 | -24.185000 |
| 3 | -33.757500 |
| 3 | -9.871333 |
| 3 | -34.783250 |
| 3 | -27.347667 |
| 3 | -16.748333 |
| 3 | -9.763333 |
| 3 | -6.127000 |
| 3 | -3.309333 |
| 3 | 7.148333 |
| 3 | 1.790571 |
| 3 | -7.076000 |
| 3 | -34.011250 |
| 3 | -28.948667 |
| 3 | -12.058000 |
| 3 | -40.115000 |
| 3 | -28.823500 |
| 3 | -28.211000 |
| 3 | -0.763833 |
